# Supplementary material for: A novel network control model for identifying personalized driver genes in cancer
Source: PLoS Comput Biol. 2019 Nov 25;15(11):e1007520. doi: 10.1371/journal.pcbi.1007520 (PMC6901264; doi:10.1371/journal.pcbi.1007520)
Supplement: S2 File — (DOCX) [file pcbi.1007520.s002.docx]

Supplementary manuscript of

**A novel network control model for identifying personalized driver genes in cancer**

Wei-Feng Guo^1^, Shao-Wu Zhang^1^*, Tao Zeng^2,5^, Yan Li^1^, Jianxi Gao^3^*, Luonan Chen^1,2,4,5^*

^1^ Key Laboratory of Information Fusion Technology of Ministry of Education, School of Automation, Northwestern Polytechnical University, Xian, 710072, China

^2^ Key Laboratory of Systems Biology, Shanghai Institutes for Biological Science, Chinese Academy Science, Shanghai 200031, China

^3^ Department of Computer Science, Rensselaer Polytechnic Institute, Troy, NY, 12180, USA

^4^ Collaborative Research Center for Innovative Mathematical Modeling, Institute of Industrial Science, University of Tokyo, Tokyo 153-8505, Japan

^5^ Shanghai Research Center for Brain Science and Brain-Inspired Intelligence, Shanghai, China

^*^ Corresponding authors: zhangsw@nwpu.edu.cn, gaoj8@rpi.edu, lnchen@sibs.ac.cn

**Supplementary note 1: The formulation of the of NCU**

Given an undirected network *G* (*V*, *E*), we generally consider the following broader class of model of [[1](#_ENREF_1)]:

（S1）

where *x_i_* denotes the state variable of the *i*-th node. The set *I_i_* is a set of neighborhood nodes of node *i*; $F_{i}(x_{i},x_{\text{Ｉ}_{\text{ｉ}}})$shows the enhancement of activity of node *I_i_*, satisfying that (i) continuous differentiability of $F_{i}(x_{i},x_{\text{Ｉ}_{\text{ｉ}}})$, that is, $F_{i}(x_{i},x_{\text{Ｉ}_{\text{ｉ}}})\in\mathrm{C}^{1}$, and (ii) dissipativity, that is, for any initial condition $x(0)$ and for a finite time $t\geq0$, the dynamical state $x(t)$ is bounded by a positive constant *C*: $\left\|x_{n}(t)\right\|\leq C$ and iii) decay condition:$\partial_{1}F_{i}(x_{i},x_{\text{Ｉ}_{\text{ｉ}}})<0$; Similar to the assumption in FVS control in directed networks[[2](#_ENREF_2), [3](#_ENREF_3)], we formalized the concept of nonlinear structural control of the undirected networks: how we chose the set of driver nodes which are injected by input signals $u$ with the minimum cost to control the system (1) from an initial attractor to a desired attractor. For the system we considered, the following theorem (Theorem 1.3 in [[2](#_ENREF_2), [3](#_ENREF_3)]) forms the basis of NCU:

*Theorem. Consider a diﬀerential equation system governed by Eq. S1 with dissipative functions$F_{i}$, and the associated undirected graph G obtained from the I_i_. We also assume functions$F_{i}$ and its derivatives to be continuous. Moreover, G can contain a self-loop only if function $F_{i}$does not satisfy the decay condition*. *Then any two solutions x and* $\mathop{x}\limits^{\text{\textasciitilde}}$ *of Eq. S1* *satisfy*

$\begin{matrix} \lim_{t\to\infty} \left(x_{J}(t)-\mathop{x}\limits^{\text{\textasciitilde}} _{J}(t)\right)\to0 \\ \Rightarrow\lim_{t\to\infty} \left(x(t)-\mathop{x}\limits^{\text{\textasciitilde}} (t)\right)\to0 \end{matrix}$ (S2)

*for all choices of nonlinearities$F_{i}$if and only if J is a feedback vertex set (FVS) of the undirected graph G.*

In Equation (S2), *x_i_*(*t*) denotes the state of the variable associated to node *i* at time *t* from initial attractor to disease attractor. The two solutions *x* and $\mathop{x}\limits^{\text{\textasciitilde}}$ denotes the state variable in the normal attractor and disease attractor of personalized state transition networks respectively. Equation S2 denotes that for all choices of nonlinearities function if and only if we drive the state of a feedback vertex set (FVS) of the undirected graph from the initial state to desired attractor (*t*→∞, *x_J_*(*t*)→$\mathop{x}\limits^{\text{\textasciitilde}} _{J}(t)$), the whole network state will be changed from an initial attractor to a desired attractor (*t*→∞, *x* (*t*)→$\mathop{x}\limits^{\text{\textasciitilde}} (t)$).

Basic on the above theorem, we formalize the concept of the Nonlinear Control of the Undirected networks (NCU), which is how we chose proper sets of driver nodes that are injected by input signal with the minimum cost to control the above equation (S1) from an initial attractor to a desired attractor. Note that the minimum FVS in undirected network *G* must exist under our assumption that each edge in undirected network is a feedback loop. Therefore our NCU aims to find minimum FVS in the undirected networks.

**Supplementary note 2:** **supplementary tables**

Table S1: Sample information in TCGA Cancer datasets. Each individual has paired samples (control sample and tumor sample)

| Number | Abbreviation | Description | Number of paired samples |
| --- | --- | --- | --- |
| 1 | BRCA | Breast invasive carcinoma | 112 |
| 2 | COAD | Colon adenocarcinoma | 50 |
| 3 | KICH | Kidney Chromophobe | 23 |
| 4 | KIRC | Kidney renal clear cell carcinoma | 72 |
| 5 | KIRP | Kidney renal papillary cell carcinoma | 31 |
| 6 | LIHC | Liver hepatocellular carcinoma | 50 |
| 7 | LUAD | Lung adenocarcinoma | 57 |
| 8 | LUSC | Lung squamous cell carcinoma | 49 |
| 9 | STAD | Stomach adenocarcinoma | 32 |
| 10 | UCEC | Uterine Corpus Endometrial Carcinoma | 23 |
| 11 | HNSC | Head and Neck Squamous Cell Carcinoma | 43 |
| 12 | PRAD | Prostate Adenocarcinoma | 52 |
| 13 | THCA | Thyroid Papillary Carcinoma | 58 |

Table S2. Concept comparisons between our NCUA and other network control methods.

| Methods | Time Complexity | Network Styles | Dynamics | Targeted state |
| --- | --- | --- | --- | --- |
| MMS | Polynomial time | Directed networks | Local nonlinear | Any |
| MDS | NP-hard | Undirected networks | Nonlinear | Any |
| DFVS | NP-hard | Directed networks | Nonlinear | Attractors |
| NCUA | NP-hard | Undirected networks | Nonlinear | Attractors |

**Supplementary note 3:** **Static model to generate the undirected scale free networks**

We use the following static model [4,5]to generate the undirected network *G* (*V*, *E*) with given exponent $\gamma$ and the given mean degree$<k>$. We start from *N* disconnected nodes indexed by integer number *i* (*i*=1,...*N*). We assign a weight $\omega_{i}=ci^{-1/(\gamma+1)}$to each node, with a real number in the range [0,1) and *c* is a constant such that

$\sum\omega_{i}=c\sum i^{-1/(\gamma+1)}=2\left\|E\right\|=<k>\left\|V\right\|$. (S3)

Two nodes *v_i_* and *v_j_* are randomly selected from the set of *N* vertices, with probability proportional to *w_i_* and *w_j_*, respectively. If they have not been connected, then connect them. Otherwise randomly choose another pair. This process is repeated until $\left\|E\right\|=\frac{<k>\left\|V\right\|}{2}$ links are created.

**Supplementary note 4: Fit of power-law distributions on personalized state transition networks**

The personalized state transition networks of different cancer data sets were fitted to power-law distributions to determine the degree exponent γ. First, we computed the connected component (CC) size of each personalized state transition network. The number of nodes in each CC on different cancer data sets is shown in **Additional file 3**. We then used each CC network to fit the power-law distribution. By following [6], let *x* represent a sequence of observations of some variable whose distribution we wish to fit as a power law. There must be some lower bound to the power-law behavior. This value is denoted as *x*_min_. Therefore, given a set containing *n* observations *x_i_* > *x*_min_ and provided that γ>1, it can be shown that the continuous distribution with the corresponding normalizing constant is

$P(x)=\frac{\gamma-1}{x_{\min}}\left[\frac{x}{x_{\min}}\right]^{-\gamma}$. (S4)

The probability that the data are drawn from a distribution that follows the power law for *x_i_* > *x*_min_ reads as

$P(x|\gamma)=\prod_{i=1}^{n}\left\{\frac{\gamma-1}{x_{\min}}\left[\frac{x_{i}}{x_{\min}}\right]^{-\gamma}\right\}$. (S5)

This expression is also called the likelihood of the data given the model. The maximum likelihood estimate (after maximization of the likelihood) for the scaling exponent reads as

$\gamma=1+n\left[\sum_{i=1}^{n} \ln\frac{x_{i}}{x_{\min}}\right]^{-1}$. (S6)

The maximum likelihood, as described above, estimates the scaling parameter γ for each possible value of *x*_min_. The Kolmogorov–Smirnov goodness-of-fit statistic KS is computed. This is done by computing the maximum distance between the cumulative distribution function (CDF) of the data and the fitted model:

$KS=\max_{x\geq x_{\min}} \left|S(x)-P_{cum}(x)\right|$, (S7)

where S(x) is the CDF of the data for the observations with value at least *x*_min_, and *P*_cum_ is the CDF for the power-law distribution that best fits the data. The estimate of *x*_min_ is determined as the value that gives the minimum value KS over all values of *x*_min_. The results for the scaling exponent of personalized state transition networks on different cancer data sets were shown in **Additional file 3**, together with the p-value. The code for calculating the degree exponent γ and the p-value of in Empirical Data are available in http://tuvalu.santafe.edu/~aaronc/powerlaws/.

**Supplementary note 5: Novel controllability findings on synthetic SF network using the NCUA**

In order to evaluate how the network parameters affect control characteristics of undirected networks, we applied our NCUA to the synthetic Scale Free (SF) networks generated by the static model [4,5]. We assumed the degree distribution of the undirected network *G* (*V*, *E*) follows$P(k)\propto k^{-\gamma}$. We first defined the fraction of the driver nodes as the controllability $n_{d}=\left\|S\right\|/\left\|V\right\|$, where$\left\|S\right\|$denotes the set of driver nodes to control the whole network and $\left\|V\right\|$denotes the number of connected nodes in the network. Then, we applied our NCUA to estimate the control characteristics on the synthetic networks. For a given γ and average degree <*k*>, 100 networks of 10,000 nodes were constructed. The fraction of the driver nodes of the NCUA on the synthetic networks was averaged over all realizations. We listed the numerical results of our NCUA for the synthetic networks in **Figure S1**. In Figure S1 (a), we showed that for γ < 2, the minimum number of input nodes increases as γ increases, while the minimum number of input nodes does not depend on the average degree <*k*>. However, if the value of γ is above 2, the minimum number of input nodes is governed by both γ and <*k*>. Furthermore, SF networks with a large value of γ above 2 or large value of <*k*> are hard to control, as shown in Figure S1 (a). In Figures S2 (b-c), we listed the minimum number of input nodes in the function of the network size for a fixed degree exponent with γ = 1.4, 1.6, and γ = 2.4, 2.6. We found that the minimum number of input nodes decreases with the increasing network size for γ < 2, while for γ > 2, the minimum number of input nodes are not affected by the network size. These results were complemented by Figure S1 (b-c), where it shows that, compared with the Erdös-Rényi random networks (ER), only fewer nodes are needed to control the entire network if the power law degree exponent γ is smaller than 3, whereas it is more difficult to controlled with a value of γ above 3. These results gave insight into heterogeneous networks (γ<3) will be easier to control with the minimum number of driver nodes than the homogeneous networks (γ>3). The more heterogeneous an SF network degree distribution is, the easier it is to control the entire system.

**Supplementary note 2: Supplementary Figures**


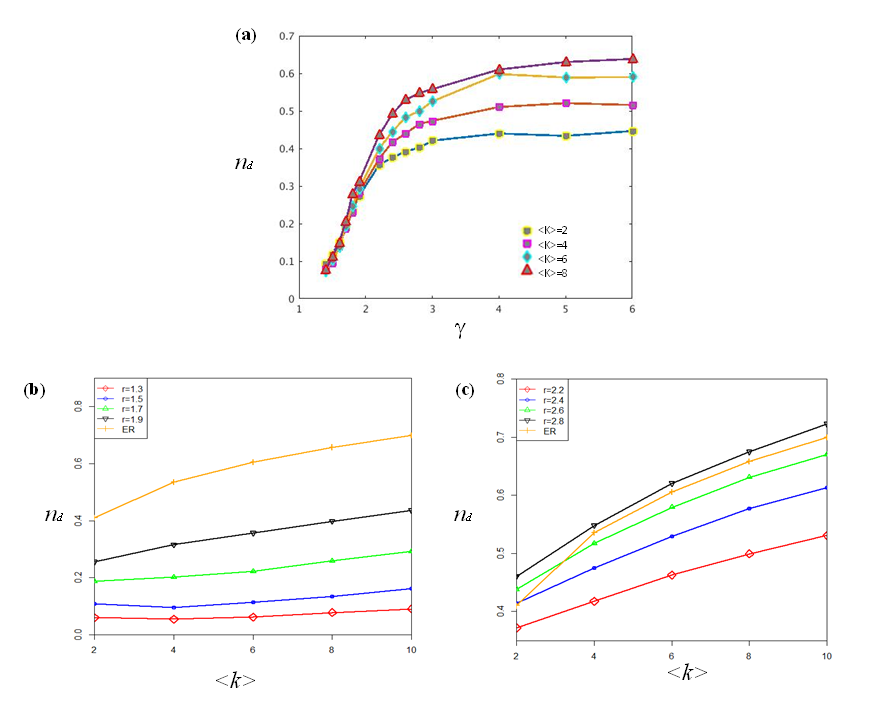


**Fig S1 Controllability of synthetic scale free undirected networks with 10,000 nodes**. All results are averaged over 100 independent realizations of the networks with 10,000 nodes. (**a**) The fraction of driver nodes for NCU control cost *n_d_* in function of the average degree <k> and the degree exponent γ for SF networks. (**b-c**) The fraction of driver nodes for NCU control cost *n_d_* in function of the average degree <k> compared with the ER networks, for the degree exponent γ larger than 2 and less than 2 respectively. It shows that compared with the ER networks, few nodes are needed to control the entire network if the power law degree exponent γ is smaller than 3, whereas more difficultly it is to be controlled, with larger value of γ than 3.


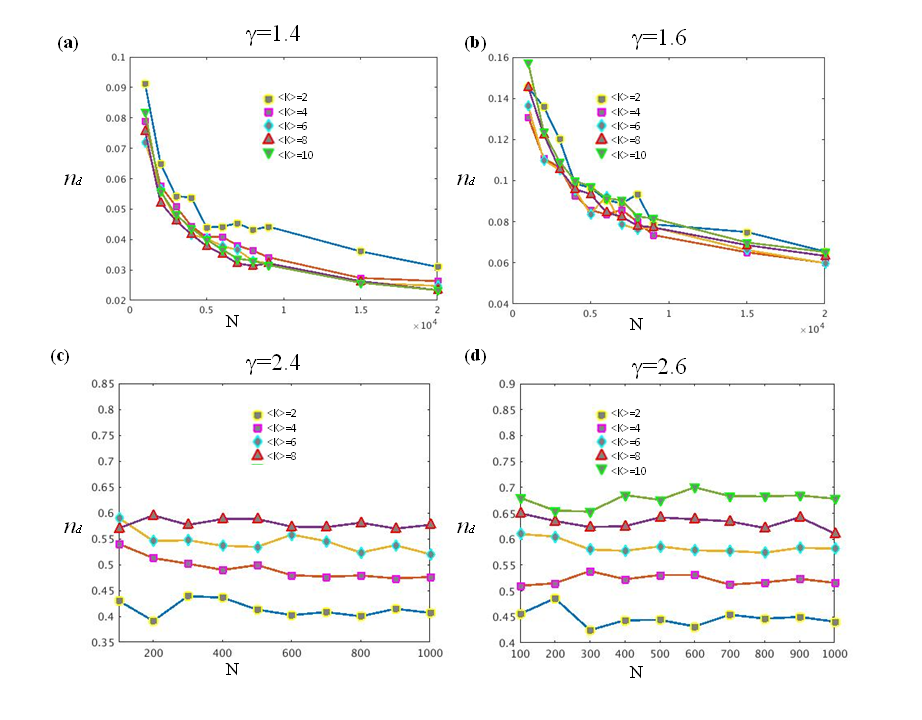


Fig S2 the minimum number of input nodes in the function of the network size for

a fixed degree exponent with γ = 1.4, 1.6 (a,b), and γ = 2.4, 2.6 (c,d).


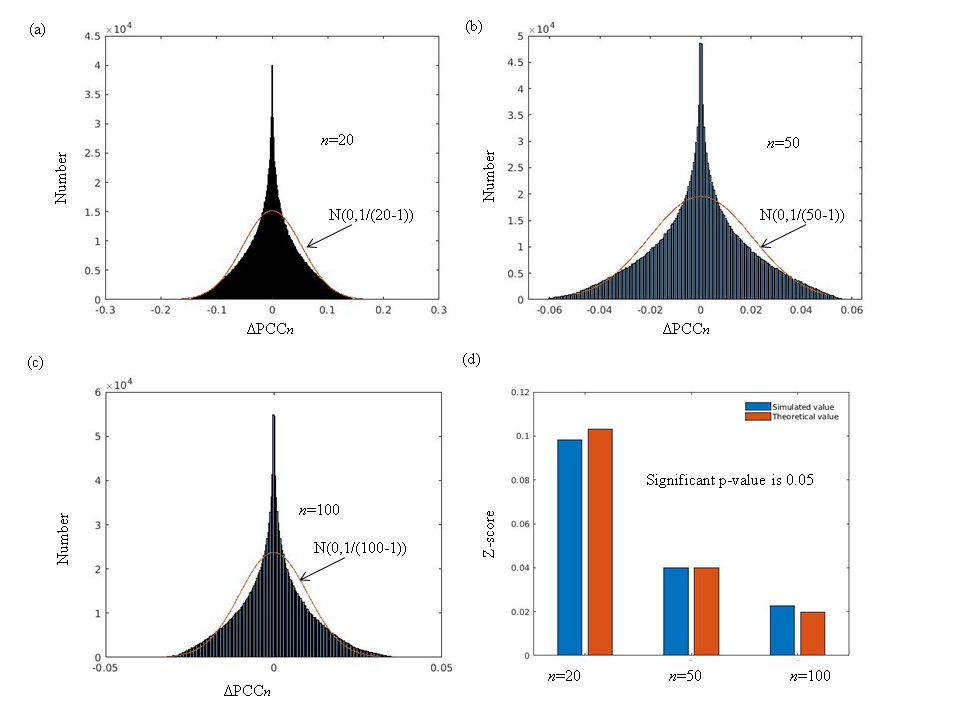


Fig S3 (a-c) The distribution of Δ*PCCn* numerically obtained by random simulation (*n*=20, 50 and 100). (d) The significant z-score of Δ*PCCn* evaluated from the distribution of the random simulation (blue color) and the theoretical distribution (red color).


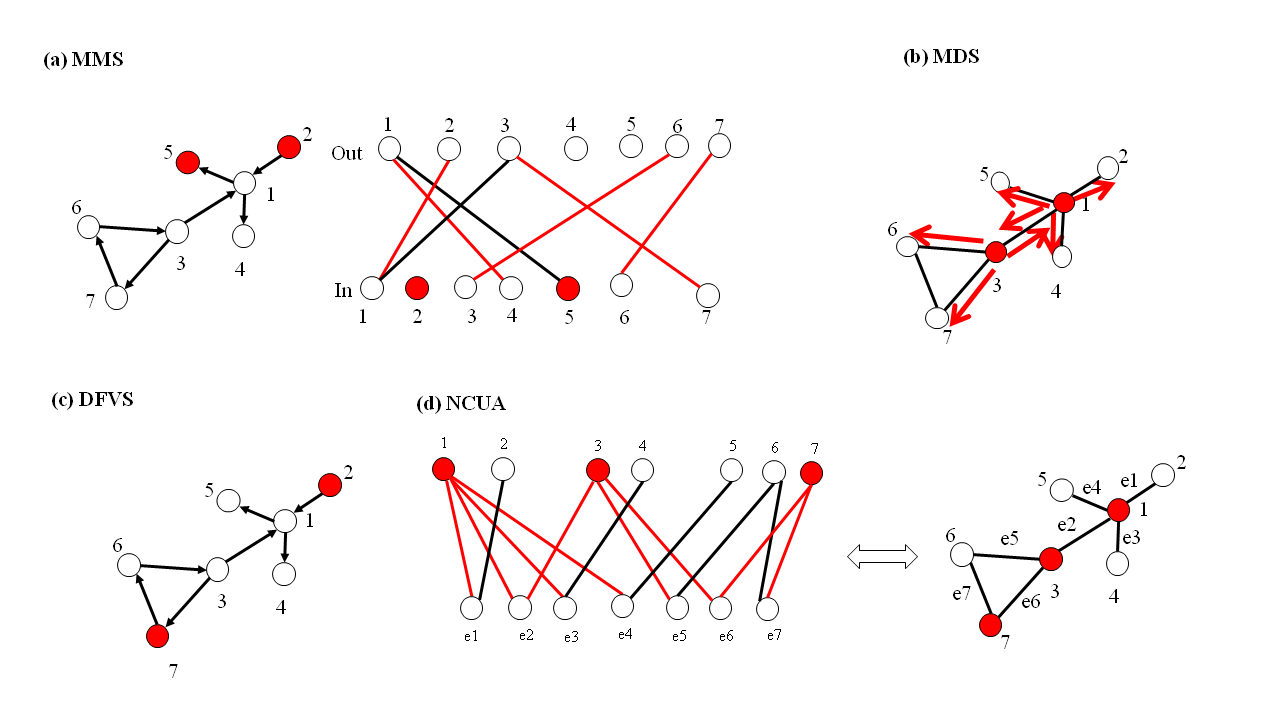
Fig S4 Schematically demonstration of MMS, MDS, DFVS and NCUA methods. (a) MMS method found the matching edges (red color edges) which results the unmatched nodes (red color nodes). These 2 nodes are considered as the driver nodes. (b) MDS method identified the minimum dominating set (red color node) as driver genes. MDS assumes that the driver node can independently control its associated edges (red color edges). (c) DFVS method identified the source node and FVS node (red color node) as the driver nodes in directed networks.（d）NCUA method assumed that each bi-directed edges in undirected networks are a feedback loop and identified 3 driver nodes (red color nodes) which can cover all the edges (feedback loop).

**References**

1. Zañudo J G T, Yang G, Albert R: Structure-based control of complex networks with nonlinear dynamic. Proceedings of the National Academy of Sciences, 2017, 114: 7234-7239.

2. Fiedler B, Kurosawa G, Saito D: Dynamics and Control at Feedback Vertex Sets. I: Informative and Determining Nodes in Regulatory Networks. Journal of Dynamics & Differential Equations 2013, 25:563-604.

3. Mochizuki A, Fiedler B, Kurosawa G, Saito D: Dynamics and control at feedback vertex sets. II: a faithful monitor to determine the diversity of molecular activities in regulatory networks. Journal of Theoretical Biology 2013, 335:130-146.

4. Liu Y, Slotine JJ, Barabasi AL: Controllability of Complex Networks. Nature, 2011, 473: 167.

5. Goh KI, Kahng B, Kim D: Universal behavior of load distribution in scale-free networks. Physical Review Letters 2001, 87:278701.

6． Clauset A, Shalizi CR, Newman MEJ: Power-Law Distributions in Empirical Data. Siam Review 2009, 51: 661-703.
