# Supplementary material for: A novel network control model for identifying personalized driver genes in cancer
Source: PLoS Comput Biol. 2019 Nov 25;15(11):e1007520. doi: 10.1371/journal.pcbi.1007520 (PMC6901264; doi:10.1371/journal.pcbi.1007520)
Supplement: Attachment — Submitted filename: Review_for_Plos_Computational_Biology_Finals.pdf [file pcbi.1007520.s010.pdf]

General comments:

This paper describes a novel method that utilizes a personalized network control model (PNC) and a structure network control method to identify the personalized driver genes by integrating the gene expression data of individual cancer patients and gene interaction network. Some components of the methodology are unnecessarily complicated and not justified well. Some datasets used in the study could be chosen more wisely. Additionally, more details are needed in order to completely evaluate the significance of the results, and a more high-level description of the proposed method would help readers to understand the intuition behind the method. The text of this paper is too theoretical and mathematical and lacks corresponding biological interpretation. It may provide valuable insight if the following concerns are addressed.

Major Comments:

a. In the last paragraph of introduction part, the authors stated that "our PNC model provides a new powerful tool for identifying the personalized driver genes of individual patients and gives novel insights for understanding tumor heterogeneity in cancer.", However, how the PNC gives novel insights for understanding tumor heterogeneity in cancer should be illustrated in the paper with at least some case studies, which is currently missing.
b. The biological intuition behind the PNC model is missing. It would be interesting if the authors can provide some examples of how their PNC model builds the personalized state transition network, what the biological meaning of the state transition network is. This could provide some biological interpretation of the PNC model.
c. Some high-level description of how the structure network control method takes advantage of the Feedback Vertex Set (FVS)-based control (FC) theory to identify the driver genes and how does it differ from related methods can help readers understand the true intellectual contribution of the paper without figuring out all the mathematical details.
d. The gene interaction network in this paper contains 11,648 genes and 211,974 interactions. There are too many edges, are there any predicated protein-protein interactions included? Are the authors used the protein-protein interactions from the STRING database? In figure1, in the step1 of paired-SSN, the toy figure of the gene interaction network is generated from the STRING database. Are those 211,974 interactions all validated by at least in-vitro experiments in the database? With 211,974 interactions, I would assume a lot of false-positive edges are included, thus leads to biased predictions. If the gene interaction network only has 10,000 very high-quality interactions, will the PNC model still work?
e. The Cancer Census Genes (CCG) and Network of Cancer Genes (NCG) are used as the gold-standard of cancer driver genes, however, are the CCG and NCG are specific for individual patients or not? If the CCG and NCG are not patient-specific driver genes, why should the authors use them as ground-truth? If the CCG and NCG are patient-specific driver genes, there is no need to use such a complex algorithm to predict the personalized driver genes.
f. The authors compared the PNC with DEG-Fold and Hub gene selection, did they compare with somatic mutations, gene amplification, and copy-number changes?
g. In the supplementary note 2, the number of paired samples is less than 80 in 12 cancer types, with such litter number, a lot of theoretical assumptions are not true due to n is small, even n=200 is very different from the assumption $n \to \infty$. The assumption regarding the distribution of PCC, the distribution of ΔPCC *and* so on are not true. The

sample-specific network theory can’t be applied in such a situation with limited numbers of patients.

h. I am not sure how the driver genes are defined?
i. There is no time-serious data, I am not sure how many states does the nodes have in the personalized state transition networks, how to determine the transition states? How to characterize the state transition of each individual in the PNC? Also in the supplementary file, in equation s2, $t \to \infty$, I am not sure what the t represents physically? As all the data are static.
j. In Table S2, the NCUA is an NP-hard problem, i.e., every time one performs the NUCA, and the result can be different.
