# Supplementary material for: A novel network control model for identifying personalized driver genes in cancer
Source: PLoS Comput Biol. 2019 Nov 25;15(11):e1007520. doi: 10.1371/journal.pcbi.1007520 (PMC6901264; doi:10.1371/journal.pcbi.1007520)
Supplement: Attachment — Submitted filename: Response_to_reviewers_0823.docx [file pcbi.1007520.s011.docx]

Responses to the Reviewers' Comments:

We thank the reviewers for giving us valuable comments, which benefit for significant improvement of our manuscript. In this document, the sentences highlighted in blue are the reviewer’s comments and the sentences highlighted in dark are our responses. The sentences highlighted in red color are the revised sentences in our manuscript.

### # Reviewer 1:

*This manuscript proposes a novel network control-based method for personalized cancer driver gene identification. This method is composed of two parts: a paired single sample network construction method to construct the personalized state transition; and a structure network control method to identify personalized driver genes. The influence of each part of the proposed method was examined. The results from the proposed method were also analyzed in terms of frequencies of personalized driver genes in different cancer types and proportion of genes with rare mutations detected in all the driver genes.*

*1 This paper lacks an in-depth and comprehensive introduction to other methods. See (https://academic.oup.com/bib/article/17/4/642/2240387) figure 1 and (https://academic.oup.com/nar/article/47/8/e45/5324448) figure 1 for a list of methods.*

**Answer：**

We thank the reviewer #1 to bring our attention to these methods, and we do agree that a comprehensive introduction is needed. After extensive literature research, we are aware of many bioinformatics tools for driver gene identification with multi-dimensional genomic data. To our best understanding, we categorized these approaches into two groups according to their significant features:

(i) The driver gene identification methods in large cohorts, such as mutation frequency-based methods and machine learning-based methods. The mutation frequency-based methods mainly identify the drive genes by finding significantly mutated genes whose mutation rates are significantly higher than the background mutation rate [1,2]. Due to the heterogeneity of tumors, constructing a reliable background mutation model is difficult, which limits the performance of frequency-based methods. On the other hand, the machine learning-based methods are suitable for any specific tasks depending on the available training data designed as pathogenic or neutral, but they have a few applications due to the probable incompleteness of their cited databases [3,4].

**(ii)** The driver gene identification methods for individual patients. These methods such as SCS [5], DawnRank [6] and OncoIMPACT [7] assume that cancer is a complex disease with many changes altered at the network level and identify personalized driver genes by integrating personalized genetic data of an individual patient and the reference gene (or protein) interaction data [8,9]. Although these methods have been successfully used for prioritizing cancer driver genes, the ultimate goal of discovering a complete catalog of driver genes indeed associated with individual patients is far from being achieved. Therefore, a new and efficient computational model or method is urgently needed to identify personalized driver genes for understanding tumor heterogeneity in cancer.

**Changes in the manuscript:**

**You may find the revised paragraph highlighted on pages 3-4 as bellow:**

Cancer is a heterogeneous disease that is driven by oncogene activations such as genetic mutation, gene amplification, chromosomal rearrangement, and transposable elements [1-3]. During tumor progression, the majority of detected altered genes are passengers that do not contribute to the oncogenic process, but a small fraction of genomic and transcriptomic altered genes are known as driver genes that modify transcriptional programs and therefore drive and sustain tumor progression from a healthy state to disease state. Many bioinformatics tools for driver gene identification with multi-dimensional genomic data have been developed recently [4-9]. To our best understanding, we categorized these approaches into two groups according to their significant features. (i) The driver gene identification methods in large cohorts, such as mutation frequency-based methods and machine learning-based methods. The mutation frequency-based methods mainly identify the drive genes by finding significantly mutated genes whose mutation rates are significantly higher than the background mutation rate [4,5]. Due to the heterogeneity of tumors, constructing a reliable background mutation model is difficult, which limits the performance of frequency-based methods. On the other hand, the machine learning-based methods can be developed for any specific tasks depending on the available training data designed as pathogenic or neutral, but they have a few applications due to the probable incompleteness of their referred databases [6, 7]. (ii) The driver gene identification methods for individual patients. These methods such as SCS [8], DawnRank [9] assume that cancer is a complex disease with many changes altered at the network level and identify personalized driver genes by integrating personalized genetic data of an individual patient and the reference gene (or protein) interaction data. Although these methods have been successfully used for prioritizing cancer driver genes, the ultimate goal of discovering a complete catalog of driver genes indeed associated with individual patients is far from being achieved. Therefore, new and efficient computational models and methods are urgently needed to identify personalized driver genes for understanding tumor heterogeneity in cancer.

*2 A major problem for this research is the performance comparison. For the comparison, only a few methods were compared (DEG-based, hub-based), whereas none of the more advanced methods (e.g., some methods in the aforementioned two figures) were compared (except for SCS, which was compared in only one dataset). Here are some methods to consider for comparison:*

*https://academic.oup.com/nar/article/47/8/e45/5324448*

*https://www.biorxiv.org/content/10.1101/456723v3*

*https://www.ncbi.nlm.nih.gov/pubmed/24516372*

*https://www.ncbi.nlm.nih.gov/pmc/articles/PMC4148527/*

*As the performance is a very important aspect of a new method, only when through comparison to current state-of-the-art methods are conducted can one draw conclusion about the value of the new method.*

**Answer：**

Beyond the first suggestion, we not only need to discuss these methods in the introduction, but also we should compare the performance of these methods with ours. We appreciate Reviewer #1 for this very constructive suggestion and offering us these critical references. Following Reviewer #1’s suggestion, we have compared our method with the current state-of-the-art and find that our method performs the best.

A list of driver genes from the Cancer Census Genes (CCG) [10-11] and the Network of Cancer Genes (NCG) [12] is used to assess the precision of the predicted driver genes. On the 13 cancer datasets, the identified cancer driver genes annotated in the CCG and NCG were adopted to compute the F-measure scores for assessing the performance of different methods. We collected 616 cancer census genes and 711 known cancer driver genes in the CCG and NCG genes.

To give a comparison of PNC with current state-of-the-art methods, our PNC selected the cancer driver genes which have high frequency (>0.8) among all the patients on each cancer data sets. From Figure 3, we found that the F-measures of PNC would be higher than 9 cancer driver genes focus methods on gene mutation data (i.e., DriverML[3], ActiveDriver[13], DriverNet [14], MutSigCV [2], OncoDriveFM [15], SCS [5], DawnRank [6] ,SSN [16] and Mutation frequency-based method) and 4 driver genes focus methods on gene expression data (i.e., DEG-FoldChange, DEG-p-value, DEG-FDR and Hub genes selection method) on 13 cancer datasets. We showed the predicted driver genes list of different methods in Additional file 4. All the above methods ran the same TCGA datasets according to the manuals.

**Changes in the manuscript:**

**You may find the revised paragraph highlighted on pages 12-13 as bellow:**

To identify personalized driver genes, we collected paired samples (a normal sample and a tumor sample) from each individual. Here we used cancer datasets which contained enough normal-disease paired samples (>20 paired samples) in TCGA for the case study. By searching TCGA, 13 cancer datasets met the requirements, i.e., the datasets for breast invasive carcinoma (BRCA), colon adenocarcinoma (COAD), kidney chromophobe (KICH) and kidney renal clear cell carcinoma (KIRC), kidney renal papillary cell carcinoma (KIRP), liver hepatocellular carcinoma (LIHC), lung adenocarcinoma (LUAD), lung squamous cell carcinoma (LUSC), stomach adenocarcinoma (STAD), uterine corpus endometrial carcinoma(UCEC), Head and Neck Squamous Cell Carcinoma (HNSC), Prostate Adenocarcinoma (PRAD) and Thyroid Papillary Carcinoma (THCA). For more information, see Table S1 of Additional File 1. On the 13 cancer datasets, the identified cancer driver genes annotated in the CCG and NCG were adopted to compute the F-measure scores (Methods) for assessing the performance of different methods. In total, we collected 616 cancer census genes and 711 known cancer genes from CCG and NCG gene lists (Additional File 2).

In the method comparisons, PNC selects the cancer driver genes which have high frequency (>0.8) among all the patients on each cancer data sets. From Figure 3, we found that the F-measures of PNC would be higher than 9 methods on gene mutation data (i.e., DriverML [7], SCS [8], DawnRank [9], ActiveDriver [22], DriverNet [23], MutSigCV [24], OncoDriveFM [25], SSN [26] and Mutation frequency method) and 4 methods on gene expression data (i.e., DEG-FoldChange, DEG-p-value, DEG-FDR and Hub genes selection method) on 13 cancer datasets. We showed the predicted driver genes list of different methods in Additional file 4. We obtained the cancer driver genes of DriverML [7], SCS[8], and SSN [26] from their provided driver genes list. We obtained the driver genes in ActiveDriver [22], OncodriverFM[25], MutSigCV[24], DriverNet[23], and DawnRank [9] from the DriverDBv2 database [34]. Besides, the DEG-FoldChange selects the personalized driver genes by calculating the fold-change between the normal sample and tumor sample (|log2(fold-change)|>1). DEG-p-value and DEG-FDR select the personalized driver genes by respectively calculating the p-value and FDR (<0.05) between a cancer tumor sample and a group of control samples. Hub genes selection method regards hub genes in the constructed network as cancer driver genes (Methods). All the above methods ran the same TCGA datasets according to their manuals.

Figure 3 The significant enrichment F-scores of PNC and other methods for identifying cancer driver genes.

*3 Please check the GitHub repo. The code can't be downloaded.*

**Answer：**

Thanks for the reviewer’s suggestion. We have uploaded the PNC code and the data resources to a new website, <https://github.com/NWPU-903PR/PNC>, which has been checked to be an efficient link.

**Changes in the manuscript:**

**You may find the revised paragraph highlighted on page 33 as bellow:**

**Availability of code and datasets**

The PNC code and the data resources used in this work can be freely downloaded from <https://github.com/NWPU-903PR/PNC>.

**Reference**

1. Tamborero D, Gonzalez-Perez A, Lopez-Bigas N (2013) OncodriveCLUST: exploiting the positional clustering of somatic mutations to identify cancer genes. Bioinformatics 29: 2238-2244.

2. Lawrence MS, Stojanov P, Polak P, Kryukov GV, Cibulskis K, et al. (2013) Mutational heterogeneity in cancer and the search for new cancer-associated genes. Nature 499: 214.

3. Han Y, Yang J, Qian X, Cheng W-C, Liu S-H, et al. (2019) DriverML: a machine learning algorithm for identifying driver genes in cancer sequencing studies. Nucleic Acids Research 47: e45-e45.

4. Luo P, Ding Y, Lei X, Wu F-X (2019) deepDriver: predicting cancer driver genes based on somatic mutations using deep convolutional neural networks. Frontiers in genetics 10.

5. Guo W-F, Zhang S-W, Liu L-L, Liu F, Shi Q-Q, et al. (2018) Discovering personalized driver mutation profiles of single samples in cancer by network control strategy. Bioinformatics 34: 1893-1903.

6. Hou JP, Ma J (2014) DawnRank: discovering personalized driver genes in cancer. Genome medicine 6: 56.

7. Bertrand D, Chng KR, Sherbaf FG, Kiesel A, Chia BK, et al. (2015) Patient-specific driver gene prediction and risk assessment through integrated network analysis of cancer omics profiles. Nucleic Acids Research 43: e44-e44.

8. Boccaletti S, Latora V, Moreno Y, Chavez M, Hwang D-U (2006) Complex networks: Structure and dynamics. Physics reports 424: 175-308.

9. Zeng T, Zhang W, Yu X, Liu X, Li M, et al. (2015) Big-data-based edge biomarkers: study on dynamical drug sensitivity and resistance in individuals. Briefings in bioinformatics 17: 576-592.

10. Futreal PA, Coin L, Marshall M, Down T, Hubbard T, et al. (2004) A CENSUS OF HUMAN CANCER GENES. Nature Reviews Cancer 4: 177-183.

11. Forbes SA, Beare D, Gunasekaran P, Leung K, Bindal N, et al. (2014) COSMIC: exploring the world's knowledge of somatic mutations in human cancer. Nucleic Acids Research 43: D805-D811.

12. Repana D, Nulsen J, Dressler L, Bortolomeazzi M, Venkata SK, et al. (2019) The Network of Cancer Genes (NCG): a comprehensive catalogue of known and candidate cancer genes from cancer sequencing screens. Genome biology 20: 1.

13. Reimand J, Bader GD (2013) Systematic analysis of somatic mutations in phosphorylation signaling predicts novel cancer drivers. Molecular systems biology 9.

14. Bashashati A, Haffari G, Ding J, Ha G, Lui K, et al. (2012) DriverNet: uncovering the impact of somatic driver mutations on transcriptional networks in cancer. Genome biology 13: R124.

15. Gonzalez-Perez A, Lopez-Bigas N (2012) Functional impact bias reveals cancer drivers. Nucleic Acids Research 40: e169-e169.

16. Liu X, Wang Y, Ji H, Aihara K, Chen L (2016) Personalized characterization of diseases using sample-specific networks. Nucleic Acids Research 44: e164-e164.

### Reviewer #2:

*This work proposed the personalized network control model (PNC) to identify the personalized driver genes. The authors firstly designed a paired single sample network construction method (Paired-SSN) to construct personalized state transition network. Then a novel structure network control method (NCUA) was presented to identify personalized driver genes. The model is interesting, but the following concerns should be addressed.*

*1.The authors mentioned that the existing methods mainly focus on cohort-level driver gene identification, and only compared their model with some simple methods in the manuscript. However, several methods aimed to identify personalized driver genes, such as DawnRank (PMID: 25177370), OncoIMPACT (PMID: 25572314), even SSN (PMID: 27596597), have been proposed. The authors should compare their model with these methods.*

**Answer：**

We appreciate Reviewer #2 for this very constructive suggestion and offering us these critical references. Following Reviewer #2’s suggestion, we have added more comparisons and discussions with SCS [1], DawnRank [2] and SSN [3] in the revised manuscript. By comparing our method with these methods, we found that our method performs the best.

A list of driver genes from the Cancer Census Genes(CCG) [4] and the Network of Cancer Genes (NCG) [5] is used to assess the precision of the predicted driver genes. On the 13 cancer datasets, the identified cancer driver genes annotated in the CCG and NCG were adopted to compute the F-measure scores for assessing the performance of different methods. We collect 616 cancer census genes and 711 known cancer driver genes in the CCG and NCG genes.

To give a comparison of PNC with current state-of-the-art methods, our PNC selected the cancer driver genes which have high frequency (>0.8) among all the patients on each cancer data sets. From Figure 3, we found that the F-measures of PNC would be higher than SCS [1], DawnRank [2] and SSN [3] on 13 cancer datasets. The predicted driver genes list of different methods are shown in Additional file 4. All these methods ran the same TCGA datasets according to their manuals.

**Changes in the manuscript:**

**You may find the revised paragraph highlighted on pages 12-13 as bellow:**

To identify personalized driver genes, we collected paired samples (a normal sample and a tumor sample) from each individual. Here we used cancer datasets which contained enough normal-disease paired samples (>20 paired samples) in TCGA for the case study. By searching TCGA, 13 cancer datasets met the requirements, i.e., the datasets for breast invasive carcinoma (BRCA), colon adenocarcinoma (COAD), kidney chromophobe (KICH) and kidney renal clear cell carcinoma (KIRC), kidney renal papillary cell carcinoma (KIRP), liver hepatocellular carcinoma (LIHC), lung adenocarcinoma (LUAD), lung squamous cell carcinoma (LUSC), stomach adenocarcinoma (STAD), uterine corpus endometrial carcinoma(UCEC), Head and Neck Squamous Cell Carcinoma (HNSC), Prostate Adenocarcinoma (PRAD) and Thyroid Papillary Carcinoma (THCA). For more information, see Table S1 of Additional File 1. On the 13 cancer datasets, the identified cancer driver genes annotated in the CCG and NCG were adopted to compute the F-measure scores (Methods) for assessing the performance of different methods. In total, we collect 616 cancer census genes and 711 known cancer genes from CCG and NCG gene lists (Additional File 2).

In the method comparisons, PNC selects the cancer driver genes which have high frequency (>0.8) among all the patients on each cancer data sets. From Figure 3, we found that the F-measures of PNC would be higher than 9 methods on gene mutation data (i.e., DriverML [7], SCS [8], DawnRank [9], ActiveDriver [22], DriverNet [23], MutSigCV [24], OncoDriveFM [25], SSN [26] and Mutation frequency method) and 4 methods on gene expression data (i.e., DEG-FoldChange, DEG-p-value, DEG-FDR and Hub genes selection method) on 13 cancer datasets. We showed the predicted driver genes list of different methods in Additional file 4. We obtained the cancer driver genes of DriverML [7], SCS[8], and SSN [26] from their provided driver genes list. We obtained the driver genes in ActiveDriver [22], OncodriverFM[25], MutSigCV[24], DriverNet[23], and DawnRank [9] from the DriverDBv2 database [34]. Besides, the DEG-FoldChange selects the personalized driver genes by calculating the fold-change between the normal sample and tumor sample (|log2(fold-change)|>1). DEG-p-value and DEG-FDR selects the personalized driver genes by respectively calculating the p-value and FDR (<0.05) between a cancer tumor sample and a group of control samples respectively. Hub genes selection method regards hub genes in the constructed network as cancer driver genes (Methods). All the above methods ran the same TCGA datasets according to their manuals.

Figure 3 The significant enrichment F-scores of PNC and other methods for identifying cancer driver genes.

*2.The authors should give more details about how to construct co-expression network for each patient.*

**Answer：**

Thanks for the reviewer’s valuable comments. It seems that the description of how to construct a co-expression network for each patient was unclear in the previous version. Following Reviewer #2’s comments, we have modified our paper and provided detailed explanations with an additional figure.

The biological system in an individul patient is generally a nonlinear dynamical system. From the dynamical viewpoint, gene expressions are variables of such a system and may be different if measured at different time points for the same patient. In contrast, it is transcriptional networks that these results in the measured gene expression pattern and determine the state transition of an individual patient in cancer development [9]. The state transition networks or transcriptional networks of an individual patient can more reliably characterize the biological system of the individual patient. The biological interpretation of personalized state transition network represents which gene pairs are involved in the disease development for each patient. Thus, as a result, it is crucial to reconstruct the personalized state transition networks with personalized genetic data (e.g., expression profiles). Currently, most of the studies for exploiting gene regulation, such as the Single Sample Network (SSN) [3] and Linear Interpolation to Obtain Network Estimates for Single Samples(LIONESS) [12], can describe the dynamic gene regulation for single tumor sample of an individual patient. However, they ignore normal sample information of an individual patient and consequently, they yield many false-positive calls for constructing personalized state transition network.

Therefore, to construct the personalized state transition network for each patient, we designed Paired-SSN method based on sample specific network theory (SSN) for characterizing significant difference of gene pairs between normal sample and tumor sample of an individual patient. For a given cancer dataset, we chose the expression data of all normal samples as the reference data and respectively constructed the co-expression network of tumor sample and normal sample with the reference data by using SSN method. Then the personalized state transition network was constructed where the nodes represent the genes and edges will exist if p-value of the gene interaction edge is less than (greater than) 0.05 in the tumor sample network but greater than (less than) 0.05 in the normal sample network. In Figure 2, we give some examples of how to apply Paired-SSN for constructing the personalized state transition network based on the expression data of two cancer patients (TCGA.G9.6499 and TCGA.EJ.7781).

**Changes in the manuscript:**

**You may find the revised paragraph highlighted on pages 10-11 as bellow:**

The biological system in an individul patient is generally a nonlinear dynamical system. From the dynamics point of view, gene expressions are variables of such a system and may be different if measured at different time points for the same patient. In contrast, it is transcriptional networks that thus result in the measured gene expression pattern and determine the state transition of an individual patient in caner development [23]. The state transition networks or transcriptional networks of an individul patient can more reliably characterize the biological system of the individual patient. The biological interpretation of personalized state transition network represents which gene pairs are involved in the disease development for each patient.

Thus it is important to reconstruct the personalized state transition networks with the personalized genetic data (e.g., expression profiles). Currently most of the studies for exploiting gene regulation, such as the Single Sample Network (SSN) [26] and Linear Interpolation to Obtain Network Estimates for Single Samples(LIONESS) [27], can describe the dynamic gene regulation for single tumor sample of an individual patient. But they ignore normal sample information of an individual patient and consequently and they yield many false-positive calls for constructing personalized state transition network. Therefore to construct the personalized state transition network for each patient, we designed Paired-SSN method based on sample specific network theory (SSN) [26] for characterizing significant difference of gene pairs between normal sample and tumor sample of an individual patient. In Figure 2, we gave some examples how to apply Paired-SSN for constructing the personalized state transition network based on the expression data of two cancer patients (TCGA.G9.6499 and TCGA.EJ.7781).

Then based on the structure of personalized state transition network, we developed NCUA method based structure based network control theory [10-13] to identify driver genes for driving the whole network state of an individual patient from the normal attractor to disease attractor. From the structure based network control respective, the personalized driver genes are the altered nodes/genes in response to input signals which can trigger the state transition of the whole gene interaction network of an individual patient. The input signals may be oncogene activation signals such as a genetic mutation, gene amplification, a chromosomal rearrangement, or transposable elements. The “controllers” in PNC model for identifying personalized driver genes are the genetic or environment factors which produce the oncogene activation signals. Therefore the biological interpretation of the PNC model is to identify personalized driver genes which can trigger the state transition of the individual system from normal attractor to disease attractor in response to oncogene activation signals.

**Figure 2 Overview of Paired-SSN for constructing personalized state transition networks.** For a given cancer patient, TCGA.G9.6499 and TCGA.EJ.7781 in Prostate Adenocarcinoma (PRAD) cancer data, we firstly chose the expression data of all normal samples in PRAD as the reference data and respectively construct the co-expression network of tumor sample (white color) and normal sample (green color) with the reference data by using SSN method. Then the personalized state transition network was constructed where the nodes represent the genes and edges denote the significant difference of gene pairs between normal sample and tumor sample of an individual patient in the disease development. Here we showed the individual specific sub-networks related with driver gene TP53 which contain its first-order neighboring genes as an example in this figure.

*3.The driver genes identified by this model were personalized. However, the existing methods they compared were aimed to identify cohort-level driver genes. The authors should describe how to summarize the personalized driver genes at the cohort-level, such as intersection, union, or some other methods.*

**Answer：**

Thanks for this valuable comment. Accordingly, we have added the descriptions of how to summarize the personalized driver genes at the cohort-level in the revised manuscript. Our PNC selected the cancer driver genes which have high frequency (>0.8) among all the patients on each cancer data sets. From Figure 3, we found that the F-measures of PNC would be higher than 9 cancer driver genes focus methods on gene mutation data (i.e., DriverML[7], ActiveDriver[8], DriverNet [9], MutSigCV [10], OncoDriveFM [11], SCS [1], DawnRank [2] , SSN [3] and Mutation frequency-based method) and 4 driver genes focus methods on gene expression data (i.e., DEG-FoldChange, DEG-p-value, DEG-FDR and Hub genes selection method) on 13 cancer datasets. All the above methods ran the same TCGA datasets according to their manuals.

**Changes in the manuscript:**

**You may find the revised paragraph highlighted on pages 12-13 as bellow:**

To identify personalized driver genes, we collected paired samples (a normal sample and a tumor sample) from each individual. Here we used cancer datasets which contained enough normal-disease paired samples (>20 paired samples) in TCGA for the case study. By searching TCGA, 13 cancer datasets met the requirements, i.e., the datasets for breast invasive carcinoma (BRCA), colon adenocarcinoma (COAD), kidney chromophobe (KICH) and kidney renal clear cell carcinoma (KIRC), kidney renal papillary cell carcinoma (KIRP), liver hepatocellular carcinoma (LIHC), lung adenocarcinoma (LUAD), lung squamous cell carcinoma (LUSC), stomach adenocarcinoma (STAD), uterine corpus endometrial carcinoma(UCEC), Head and Neck Squamous Cell Carcinoma (HNSC), Prostate Adenocarcinoma (PRAD) and Thyroid Papillary Carcinoma (THCA). For more information, see Table S1 of Additional File 1. On the 13 cancer datasets, the identified cancer driver genes annotated in the CCG and NCG were adopted to compute the F-measure scores (Methods) for assessing the performance of different methods. In total, we collect 616 cancer census genes and 711 known cancer genes from CCG and NCG gene lists (Additional File 2).

In the method comparisons, PNC selects the cancer driver genes which have high frequency (>0.8) among all the patients on each cancer data sets. From Figure 3, we found that the F-measures of PNC would be higher than 9 methods on gene mutation data (i.e., DriverML [7], SCS [8], DawnRank [9], ActiveDriver [22], DriverNet [23], MutSigCV [24], OncoDriveFM [25], SSN [26] and Mutation frequency method) and 4 methods on gene expression data (i.e., DEG-FoldChange, DEG-p-value, DEG-FDR and Hub genes selection method) on 13 cancer datasets. We showed the predicted driver genes list of different methods in Additional file 4. We obtained the cancer driver genes of DriverML [7], SCS[8], and SSN [26] from their provided driver genes list. We obtained the driver genes in ActiveDriver [22], OncodriverFM[25], MutSigCV[24], DriverNet[23], and DawnRank [9] from the DriverDBv2 database [34]. Besides, the DEG-FoldChange selects the personalized driver genes by calculating the fold-change between the normal sample and tumor sample (|log2(fold-change)|>1). DEG-p-value and DEG-FDR selects the personalized driver genes by respectively calculating the p-value and FDR (<0.05) between a cancer tumor sample and a group of control samples respectively. Hub genes selection method regards hub genes in the constructed network as cancer driver genes (Methods). All the above methods ran the same TCGA datasets according to their manuals.

*4.In P16, the authors said “in the KIRP cancer dataset, NCUA on Paired-SSN had higher F-measure than SSN method......”. But it was lower in Figure 3(a).*

**Answer：**

Thanks for the reviewer’s suggestion. We have carefully checked the improper statements and the typesetting of equations and variables, and made corrections in this revised manuscript.

**Changes in the manuscript:**

**You may find the revised paragraph highlighted on pages 14 as bellow:**

Based on the personalized state transition networks constructed by LIONESS, SSN and Paired-SSN respectively, we applied the NCUA method for identifying the personalized driver genes (Figure 4 (a)). From Figure 4 (a), we found that considering the CCG and NCG genes as current gold-standard of cancer driver genes, the F-measures of network control methods (i.e. NCUA) would be higher when Paired-SSN networks rather than LIONESS and SSN were used; and Paired-SSN obviously improve original SSN. Therefore, in contrast to other personalized state transition network inferred by other traditional methods such as SSN and LIONESS, Paired-SSN can include more information of individual patients and is suitable for the construction of personalized state transition networks in this work.

*5.The authors should give the full name or brief description about LIONESS when it was firstly appeared in P11.*

**Answer：**

Thanks for the reviewer’s suggestion. We have added the full name of LIONESS when it was firstly appeared in the revised manuscript.

**Changes in the manuscript:**

**You may find the revised sentences highlighted on pages 13 as bellow:**

Meanwhile, LIONESS (Linear Interpolation to Obtain Network Estimates for Single Samples) [27] constructs the personalized state transition networks by calculating the edge statistical significance between all tumor samples and the tumor samples without a given single sample (Methods).

**Reference**

1. Guo W-F, Zhang S-W, Liu L-L, Liu F, Shi Q-Q, et al. (2018) Discovering personalized driver mutation profiles of single samples in cancer by network control strategy. Bioinformatics 34: 1893-1903.

2. Hou JP, Ma J (2014) DawnRank: discovering personalized driver genes in cancer. Genome medicine 6: 56.

3. Liu X, Wang Y, Ji H, Aihara K, Chen L (2016) Personalized characterization of diseases using sample-specific networks. Nucleic Acids Research 44: e164-e164.

4. Futreal PA, Coin L, Marshall M, Down T, Hubbard T, et al. (2004) A CENSUS OF HUMAN CANCER GENES. Nature Reviews Cancer 4: 177-183.

5. Repana D, Nulsen J, Dressler L, Bortolomeazzi M, Venkata SK, et al. (2019) The Network of Cancer Genes (NCG): a comprehensive catalogue of known and candidate cancer genes from cancer sequencing screens. Genome biology 20: 1.

6. Chung I-F, Chen C-Y, Su S-C, Li C-Y, Wu K-J, et al. (2015) DriverDBv2: a database for human cancer driver gene research. Nucleic Acids Research 44: D975-D979.

7. Han Y, Yang J, Qian X, Cheng W-C, Liu S-H, et al. (2019) DriverML: a machine learning algorithm for identifying driver genes in cancer sequencing studies. Nucleic Acids Research 47: e45-e45.

8. Reimand J, Bader GD (2013) Systematic analysis of somatic mutations in phosphorylation signaling predicts novel cancer drivers. Molecular systems biology 9.

9. Bashashati A, Haffari G, Ding J, Ha G, Lui K, et al. (2012) DriverNet: uncovering the impact of somatic driver mutations on transcriptional networks in cancer. Genome biology 13: R124.

10. Lawrence MS, Stojanov P, Polak P, Kryukov GV, Cibulskis K, et al. (2013) Mutational heterogeneity in cancer and the search for new cancer-associated genes. Nature 499: 214.

11. Gonzalez-Perez A, Lopez-Bigas N (2012) Functional impact bias reveals cancer drivers. Nucleic Acids Research 40: e169-e169.

12. Kuijjer ML, Tung MG, Yuan G, Quackenbush J, Glass K (2019) Estimating sample-specific regulatory networks. iScience 14: 226-240.

### Reviewer #3:

*General comments:*

*This paper describes a novel method that utilizes a personalized network control model (PNC) and a structure network control method to identify the personalized driver genes by integrating the gene expression data of individual cancer patients and gene interaction network. Some components of the methodology are unnecessarily complicated and not justified well. Some datasets used in the study could be chosen more wisely. Additionally, more details are needed in order to completely evaluate the significance of the results, and a more high-level description of the proposed method would help readers to understand the intuition behind the method. The text of this paper is too theoretical and mathematical and lacks corresponding biological interpretation. It may provide valuable insight if the following concerns are addressed.*

*Major Comments:*

*a. In the last paragraph of introduction part, the authors stated that “our PNC model provides a new powerful tool for identifying the personalized driver genes of individual patients and gives novel insights for understanding tumor heterogeneity in cancer.”, However, how the PNC gives novel insights for understanding tumor heterogeneity in cancer should be illustrated in the paper with at least some case studies, which is currently missing.*

**Answer：**

We thank Reviewer #3 for recognizing the novelty of our method and promoting us to emphasize the novelty of our method by additional case studies. To illustrate the tumor heterogeneity in various types of cancer, we used the individual-specific sub-networks related with known driver gene *TP53*, which contain its first-order neighboring genes as an example. By integrating the sub-networks of patients on each cancer data set, we obtained the statistic information of individual-specific sub-networks of *TP53* including frequency as personalized driver genes, the SNVs mutation frequency, mean differential expression fold change (the absolute value of log2 fold change between normal expression data and tumor expression data) and mean degree in individual-specific sub-networks (Table 1). From Table 1, we can see that: i) although TP53 has different mutation frequency in multiple cancer data sets, PNC identified TP53 having high driver frequency in most of cancer data sets. This result demonstrates that PNC predicts individual driver genes for cancer solely based on gene expression without DNA sequence information. ii) TP53 has low differential expression fold change (<1) in some cancer data sets (COAD, KIRP, LUSC, STAD, THCA, and PRAD) but has high mean network degree in all cancer data sets. This result demonstrates that even when the driver genes are hidden in transcription profiles but can be explored by their network and structural characteristics.

Next, we computed the p-value of the individual-specific *TP53* sub-networks enriching in the KEGG pathways by using the hypergeometric test (Methods). By integrating the individual-specific pathways in 13 cancer data sets, we obtained the cancer-related pathways with its patient frequency in multiple cancer data sets. We found that 44 pathways which appeared in all 13 cancer data sets and among them 20 pathways (45.45%) were reported to be related to cancer in previous biological observations. In Figure 7, we showed the patient frequency value of these 20 pathways in 13 cancer data sets. From Figure 7, we can see that i) *cell cycle pathway* is enriched in most of the patients in 12 cancer data sets (except COAD), demonstrating that TP53 regulates cell cycle genes in most of the cancer patients; ii) *Adherens junction pathway* is enriched in most of the patients (69.38%) for LUSC cancer data set while it is enriched in small number of patients (<50%) for other cancer data sets. These results demonstrate that tumor heterogeneity for the enriched pathways varies in different cancer data sets. Therefore, our PNC gives novel insights into understanding the tumor heterogeneity in cancer.

**Changes in the manuscript:**

**You may find the revised paragraph highlighted on pages 19-21 as bellow:**

To illustrate the above demonstrated tumor heterogeneity in various types of cancer, we used the individual specific sub-networks related with driver gene *TP53* and its first-order network neighboring genes as an example. By integrating the sub-networks of patients on each cancer data set, we obtained the statistic information of individual specific sub-networks of *TP53* including frequency as personalized driver genes, the SNVs mutation frequency, mean differential expression fold change (the absolute value of log2 fold change between normal expression data and tumor expression data), and mean network degree in individual specific sub-networks (Table 1). We can see that i) although *TP53* has different mutation frequency in multiple cancer data sets, PNC identified *TP53* having high driver frequency in most of cancer data sets. This result demonstrates that PNC is able to predict individual driver genes for cancer solely based on gene expression without DNA sequence information; ii) *TP53* has low differential expression fold change (|log2(fold-change)|<1) in some cancer data sets (COAD, KIRP, LUSC, STAD, THCA and PRAD) but has high average network degree so as to be hub gene. This result demonstrates that even when the candidate driver genes are hidden in transcription profiles, they can still be explored by their network and structural characteristics, e.g. by PNC.

Next we computed the p-value of the individual-specific *TP53* sub-networks enriching in the KEGG pathways by using the hyper-geometric test (Methods). By integrating the individual-specific pathways in 13 cancer data sets, we obtain the cancer pathways with its patient frequency in multiple cancer data sets. All enriched pathways in 13 cancer data sets are listed in Additional file 5. We found that 44 pathways which appeared in all 13 cancer data sets and 45.45% of them were reported to be related with cancer in previous biological observations. The detail results were listed in Additional file 6. In Figure 7 we showed the patient frequency value of these confident common pathways in 13 cancer data sets. We can see that i) most of these pathways are observed in only a little fraction of the patients in COAD; ii) *cell cycle pathway* is enriched in most of patients in 12 cancer data sets (except COAD), demonstrating that cell cycle genes are regulated by TP53 in most of cancer patients; ii) *Adherens junction pathway* are enriched in most of patients (69.38%) for LUSC cancer data set while it is enriched in small number of patients (<50%) for other cancer data sets. These results demonstrate that there are even different tumor heterogeneity for the same pathway in different cancer types. Therefore, our PNC is capable to reveal some novel insights into understanding the tumor heterogeneity in cancer.

**Table 1 The statistic information of individual specific sub-networks of TP53 in cancer.**

| Cancer | Driver frequency | Mutation Frequency | Mean Fold change | Mean degree |
| --- | --- | --- | --- | --- |
| BRCA | 1 | 0.3111 | 9.5347 | 380.32 |
| COAD | 0.92 | 0.4870 | 0.2095 | 38.98 |
| LUAD | 1 | 0.4608 | 10.8065 | 387.49 |
| LUSC | 1 | 0.8202 | 5.3138 | 339.75 |
| KICH | 1 | 0.3333 | 23.9574 | 400.52 |
| KIRC | 1 | 0.0160 | 1.0044 | 176.08 |
| KIRP | 1 | 0.0248 | 0.1905 | 243.41 |
| LIHC | 1 | 0.3181 | 8.8303 | 389.20 |
| UCEC | 1 | 0.2862 | 11.3241 | 359.39 |
| STAD | 1 | 0.4775 | 0.0036 | 224.71 |
| THCA | 1 | 0.0074 | 0.3888 | 147.87 |
| PRAD | 0.9423 | 0.0692 | 0.6188 | 147.40 |
| HNSC | 1 | 0.7347 | 1.2537 | 152.72 |

**Figure 7** Heat map of the patient frequency (different colors) of these 20 intersected pathways in 13 cancer data sets which was previous reported in other literatures.

*b. The biological intuition behind the PNC model is missing. It would be interesting if the authors can provide some examples of how their PNC model builds the personalized state transition network, what the biological meaning of the state transition network is. This could provide some biological interpretation of the PNC model.*

**Answer：**

Thanks for the reviewer’s valuable comments. It seems that the biological interpretation of the PNC model was unclear in the previous version. Following Reviewer #3’s value comments, we have modified our paper and provided detailed biological intuition behind the PNC model.

The biological system in an individual patient is generally a nonlinear dynamical system. From the dynamical viewpoint, gene expressions are variables of such a system and may be different if measured at different time points for the same patient. In contrast, it is transcriptional networks that these results in the measured gene expression pattern and determine the state transition of an individual patient in cancer development [19]. The state transition networks or transcriptional networks of an individual patient can more reliably characterize the biological system of the individual patient. The biological interpretation of personalized state transition network represents which gene pairs are involved in the disease development for each patient. Thus, as a result, it is crucial to reconstruct the personalized state transition networks with the personalized genetic data (e.g., expression profiles). Currently, most of the studies for exploiting gene regulation, such as the Single Sample Network (SSN) [1] and Linear Interpolation to Obtain Network Estimates for Single Samples(LIONESS) [2], can describe the dynamic gene regulation for a single sample of an individual patient. However, they ignore normal sample information of an individual patient and consequently, they yield many false-positive calls for constructing personalized state transition network. Therefore, to build the personalized state transition network for each patient, we designed Paired-SSN method based on sample-specific network theory (SSN) for characterizing significant difference of gene pairs between normal sample and tumor sample of an individual patient. In Figure 2, we gave some examples of how to apply Paired-SSN for constructing the personalized state transition network based on the expression data of two cancer patients (TCGA.G9.6499 and TCGA.EJ.7781).

Then based on the structure of personalized state transition network, we developed NCUA method based on structure-based network control theory to identify driver genes for driving the whole network state of an individual patient from the normal attractor to disease attractor. From the structure-based network control respective, the personalized driver genes are the altered nodes/genes in response to input signals, which can trigger the state transition of the whole gene interaction network of an individual patient. The input signals may be oncogene activation signals such as a genetic mutation, gene amplification, a chromosomal rearrangement, or transposable elements. The “controllers” in PNC model for identifying personalized driver genes are the genetic or environmental factors which produce the oncogene activation signals. Therefore, the biological interpretation of the PNC model is to identify personalized driver genes which can trigger the state transition of the individual system from normal attractor to disease attractor in response to oncogene activation signals.

**Changes in the manuscript:**

**You may find the revised paragraph highlighted on pages 10-11 as bellow:**

The biological system in an individul patient is generally a nonlinear dynamical system. From the dynamics point of view, gene expressions are variables of such a system and may be different if measured at different time points. In contrast, it is transcriptional networks that thus result in the measured gene expression pattern and determine the state transition of an individual patient in caner development [23]. The state transition networks or transcriptional networks of an individul patient can more reliably characterize the biological system of the individual patient.The biological interpretation of personalized state transition network represents which gene pairs are involved in the disease development for each patient.

Thus it is important to reconstruct the personalized state transition networks with the personalized genetic data (e.g., expression profiles). Currently most of the studies for exploiting gene regulation, such as the Single Sample Network (SSN) [26] and Linear Interpolation to Obtain Network Estimates for Single Samples(LIONESS) [27], can describe the dynamic gene regulation for single tumor sample of an individual patient. But they ignore normal sample information of an individual patient and consequently and they yield many false-positive calls for constructing personalized state transition network. Therefore to construct the personalized state transition network for each patient, we designed Paired-SSN method based on sample specific network theory (SSN) [26] for characterizing significant difference of gene pairs between normal sample and tumor sample of an individual patient. In Figure 2, we gave some examples how to apply Paired-SSN for constructing the personalized state transition network based on the expression data of two cancer patients (TCGA.G9.6499 and TCGA.EJ.7781).

Then based on the structure of personalized state transition network, we developed NCUA method based structure based network control theory [10-13] to identify driver genes for driving the whole network state of an individual patient from the normal attractor to disease attractor. From the structure based network control respective, the personalized driver genes are the altered nodes/genes in response to input signals which can trigger the state transition of the whole gene interaction network of an individual patient. The input signals may be oncogene activation signals such as a genetic mutation, gene amplification, a chromosomal rearrangement, or transposable elements. The “controllers” in PNC model for identifying personalized driver genes are the genetic or environment factors which produce the oncogene activation signals. Therefore the biological interpretation of the PNC model is to identify personalized driver genes which can trigger the state transition of the individual system from normal attractor to disease attractor in response to oncogene activation signals.

**Figure 2 Overview of Paired-SSN for constructing personalized state transition networks.** For a given cancer patient, TCGA.G9.6499 and TCGA.EJ.7781 in Prostate Adenocarcinoma (PRAD) cancer data, we firstly chose the expression data of all normal samples in PRAD as the reference data and respectively construct the co-expression network of tumor sample (white color) and normal sample (green color) with the reference data by using SSN method. Then the personalized state transition network was constructed where the nodes represent the genes and edges denote the significant difference of gene pairs between normal sample and tumor sample of an individual patient in the disease development. Here we showed the individual specific sub-networks related with driver gene TP53 which contain its first-order neighboring genes as an example in this figure.

*c. Some high-level description of how the structure network control method takes advantage of the Feedback Vertex Set (FVS)-based control (FC) theory to identify the driver genes and how does it differ from related methods can help readers understand the true intellectual contribution of the paper without figuring out all the mathematical details.*

**Answer：**

Thanks for the constructive suggestions of Reviewer #3. We do agree that it is crucial to show how our method differs from the current state-of-the-art methods, which definitely helps readers understand the significant contribution of this paper without knowing the details of the math. We appreciate Reviewer #3 for this valuable comment. Since many real complex systems, especially the biological systems, usually, have hundreds and thousands of dynamical components so that the classical control theory is prohibited [9].

The biological system is generally a nonlinear dynamical system. From the dynamics point of view, gene expressions are variables of such a system and may be different if measured at different time points. In contrast, it is transcriptional networks that these results in the measured gene expression pattern of an individual patient [19]. Indeed, transcriptional networks are composed of numerous nodes linked via a complex set of interactions, and the dynamical behavior is controlled by a few driver nodes [9]. To be specific, human fibroblasts could be reprogrammed into an induced pluripotent stem cell (iPSCs), via overexpression of four transcription factor POU5F1, SOX2, KLF4, and MYC [30].

It is necessary to investigate the relationship between the structure and the dynamics of complex systems [8,9]. Recently, structure-based network control approaches, which only should know whether there are edges or not in the state transition networks, connect the structure and the dynamics of complex systems, enabling us to steer the states of complex systems to the desired states within finite time [8,9]. The structure-based network control approaches are useful for complex biological networks because we usually know structures of them but do not know the specific interaction strengths. In past decades, scientists have been studying the structure-based network control of systems with both linear dynamics and nonlinear dynamics [3-5]. Recently, the FC control method [6,7] has been successfully applied to large scale complex biological networks with known network topologies but without knowing the nonlinear dynamical equations that govern their state transition. It only requires the functional form of the governing equations to satisfy some continuous, dissipative, and decaying properties. To drive the state of a network to any one of its naturally occurring end states (i.e., dynamical attractors), FC needs to manipulate a set of nodes (i.e., the FVS) that intersects every feedback loop in the network. Under the FC framework, DFVS is original designed to study the structural control problem of directed networks, by selecting a minimum set of driver nodes to render a directed network structural controllable, which has been recently discussed [8]. The selection of driver nodes depends on the directionality of the given networks (directed or undirected networks). Motivated by this fact, we developed a graphic-theoretic algorithm called Nonlinear Control of Undirected network Algorithm (NCUA) under the nonlinear FC framework for determining the minimum driver nodes in undirected networks.

To better understand NCUA, we gave the concept comparisons including the network types and targeted states and time complexity and network dynamics between our NCUA and other network control method including MMS, MDS, and DFVS (Table S2 of Additional file 1). We summarized some key points of different structure network control methods as follow: i) The MMS based control methods investigate the controllability of directed structural networks with linear or local nonlinear dynamics through a minimum set of input nodes and they only give an incomplete view of the network control properties for a system with nonlinear dynamics; ii) MDS control method studies the controllability of undirected networks by assuming that each driver node in the MDS model can control its associated edges independently in the undirected networks. Since MDS works with the strong assumption that the controllers can control its outgoing links independently, it requires higher costs in many kinds of networks which may underestimate the structural control analysis of undirected networks; iii) NCUA and DFVS study the structural network control of undirected and directed networks respectively based on the framework of FC. Therefore NCUA and DFVS methods ultimately depict the structure-based network control of the large-scale system with nonlinear dynamics from the respective of FC. Since the FC control method [6,7] assumes that the functional form of the governing equations must satisfy some continuous, dissipative, and decaying properties, DFVS and NCUA may be only suitable some specialized nonlinear systems. In Figure S4, we used an example to intuitively explain the difference between the NCUA and MMS, MDS, and DFVS.

Figure S4 Schematically demonstration of MMS, MDS, DFVS and NCUA methods. (a) MMS method found the matching edges (red color edges) which results the unmatched nodes (red color nodes). These 2 nodes are considered as the driver nodes. (b) MDS method identified the minimum dominating set (red color node) as driver genes. MDS assumes that the driver node can independently control its associated edges (red color edges). (c) DFVS method identified the source node and FVS node (red color node) as the driver nodes in directed networks. (d) NCUA method assumed that each bi-directed edges in undirected networks are a feedback loop and identified 3 driver nodes (red color nodes) which can cover all the edges (feedback loop).

**Changes in the manuscript:**

**You may find the revised paragraph highlighted on pages 30-31 as bellow:**

To easier understand NCUA, we gave the concept comparisons including the network types and targeted states and time complexity and network dynamics between our NCUA and other network control method including MMS [11-13], MDS [19] and DFVS [18] (Table S2 of Additional file 1). In Figure S4, we used an example to intuitively explain the difference between the NCUA and MMS, MDS and DFVS. We summarized some key points of different structure network control methods as follow:

i) The MMS based control methods investigate the controllability of directed structural networks with linear or local nonlinear dynamics through a minimum set of input nodes and they only give an incomplete view of the network control properties for a system with nonlinear dynamics.

ii) MDS control method studies the controllability of undirected networks by assuming that each driver node in the MDS model can control its associated edges independently in the undirected networks. Since MDS works with the strong assumption that the controllers can control its outgoing links independently, it requires higher costs in many kinds of networks which may underestimate the structural control analysis of undirected networks;

iii) NCUA and DFVS study the structural network control of undirected and directed networks respectively based on the framework of FC. Therefore NCUA and DFVS methods ultimately depict the structure-based network control of the large-scale system with nonlinear dynamics from the respective of FC. Since the FC control method [16,17] assumes that the functional form of the governing equations must satisfy some continuous, dissipative, and decaying properties, DFVS and NCUA may be only suitable some specialized nonlinear systems.

*d. The gene interaction network in this paper contains 11,648 genes and 211,974 interactions. There are too many edges, are there any predicated protein-protein interactions included? Are the authors used the protein-protein interactions from the STRING database? In figure1, in the step1 of paired-SSN, the toy figure of the gene interaction network is generated from the STRING database. Are those 211,974 interactions all validated by at least in-vitro experiments in the database? With 211,974 interactions, I would assume a lot of false-positive edges are included, thus leads to biased predictions. If the gene interaction network only has 10,000 very high-quality interactions, will the PNC model still work?*

**Answer：**

Thanks for the reviewer’s valuable comments. We have to note that there are many edges in our network, and these edges include gene-interactions from multiple high-quality sources such as protein interactions, gene co-expressions, TF-target interactions, protein domain interactions, and text-mined interactions. In figure 1 of our original manuscript, the toy figure of the gene interaction network is not proper, causing Reviewer #3 to misunderstand that the gene interaction network was generated from the STRING database. We have replaced it with another illustrative figure of the gene interaction network in the revised Figure 1. Although these interactions are not from STRING, instead of from the website http://bioen-compbio.bioen.illinois.edu/DawnRank/[14], the reference gene interaction network was validated in a variety of data sources, including MEMo [10], Reactome [11], the NCI-Nature Curated PID [12], and KEGG[13].

Furthermore, to estimate the effect of the reference network adopted in PNC, we also used other gene interaction networks as the reference networks, including gene interaction networks collected in reference [15] (Network 2), and reference [16] (Network 3). Following Reviewer #3’s question, we also tested the performance of our method on gene interaction networks from STRING data set (<https://string-db.org/>) whose edge scores are higher than 900 (Network 4) and a simulated network (Network 5) by choosing the top 10, 000 high scores interactions from STRING data set. The performance of PNC with five kinds of gene interaction networks on the 13 cancer datasets are shown in **Figure 4 (c)**. From Figure 4 (c), we can see that Network 1 with 211,974 interactions with more complete and higher quality gene interaction information would improve PNC's prediction power. Furthermore, PNC on Network 4 with 10,000 high-quality interactions, has lower performance compared Network 5 with the complete gene interaction information from STRING data set. This result highlights that if the gene interaction network only has 10,000 high-quality interactions, the incompleteness of reference networks would decrease PNC's prediction power.

**Changes in the manuscript:**

**You may find the revised paragraph highlighted on pages 15-16 as bellow:**

Furthermore, to estimate the effect of the reference network adopted in PNC, we also used other gene interaction networks as the reference networks, including gene interaction network collected in reference [14] (Network 2), and reference [35] (Network 3) and gene interaction networks from STRING data set (https://string-db.org/) (Network 4). By integrating multiple types of datasets, including Reactome [31], NCI-Nature Curated PID [32] and Kyoto Encyclopedia of Genes and Genomes [33], the Network 2 consists of 5959 genes and 108,281 edges. For the Network 3, 6513 genes and 19,955 synthetic lethal gene pairs were collected the Synthetic Lethality Database (https://omictools.com/synlethdb-tool). By choosing the edge scores higher than 900 from STRING, the Network 4 consists of 12,272 proteins (genes) and 505,116 edges with confidence scores. We also tested the performance of our method on a simulated network with by choosing the top 10, 000 high quality interactions from STRING data set (Network 5). The performance of PNC with five kinds of gene interaction networks on the 13 cancer datasets were shown in **Figure 4 (c)**. From Figure 4 (c), we can see that Network 1 with 211,974 interactions with more complete and higher quality gene interaction information would improve PNC's prediction power.

**Figure 4** The performance evaluation of different single sample network construction methods and network control methods. (a) The significant enrichment F-scores of NCUA on personalized state transition networks constructed by Paired-SSN (the first step of our PNC), SSN and LIONESS. (b)The enrichment F-scores of NCUA (the second step of our PNC) and other structure network control methods (MMS and MDS and DFVS) on the paired SSN networks (the first step of our PNC) in the list of CCG and NCG genes. (c) The enrichment F-scores of PNC with gene interaction network used in this work (Network 1) and reference [14] (Network 2), and reference [35] (Network 3) and the gene interaction network from STRING data set (scores>900) (Network 4) and the gene interaction network with top 10000 high scores from STRING data set (Network 5).

*e. The Cancer Census Genes (CCG) and Network of Cancer Genes (NCG) are used as the gold-standard of cancer driver genes, however, are the CCG and NCG are specific for individual patients or not? If the CCG and NCG are not patient-specific driver genes, why should the authors use them as ground-truth? If the CCG and NCG are patient-specific driver genes, there is no need to use such a complex algorithm to predict the personalized driver genes.*

**Answer：**

Reviewer #3 is correct and the Cancer Census Genes (CCG) and Network of Cancer Genes (NCG) should be used as the gold-standard of cancer driver genes. We do agree that it is not proper to use the CCG and NCG as ground-truth for evaluating patient-specific driver genes directly in the previous version of our manuscript. Therefore the identified cancer driver genes annotated in the CCG and NCG were adopted to compute the F-measure scores for assessing the performance of different methods. To give a comparison of PNC with current state-of-the-art methods, our PNC selected the cancer driver genes which have high frequency (>0.8) among all the patients on each cancer data sets. Furthermore, in Figure 3, we gave a comparison of PNC with current state-of-the-art methods on 13 cancer data sets. From Figure 3, we found that the F-measures of PNC would be higher than current state-of-the-art methods on 13 cancer datasets. We appreciate Reviewer #3 for these valuable quetions.

**Changes in the manuscript:**

**You may find the revised paragraph highlighted on pages 12-13 as bellow:**

To identify personalized driver genes, we collected paired samples (a normal sample and a tumor sample) from each individual. Here we used cancer datasets which contained enough normal-disease paired samples (>20 paired samples) in TCGA for the case study. By searching TCGA, 13 cancer datasets met the requirements, i.e., the datasets for breast invasive carcinoma (BRCA), colon adenocarcinoma (COAD), kidney chromophobe (KICH) and kidney renal clear cell carcinoma (KIRC), kidney renal papillary cell carcinoma (KIRP), liver hepatocellular carcinoma (LIHC), lung adenocarcinoma (LUAD), lung squamous cell carcinoma (LUSC), stomach adenocarcinoma (STAD), uterine corpus endometrial carcinoma(UCEC), Head and Neck Squamous Cell Carcinoma (HNSC), Prostate Adenocarcinoma (PRAD) and Thyroid Papillary Carcinoma (THCA). For more information, see Table S1 of Additional File 1. On the 13 cancer datasets, the identified cancer driver genes annotated in the CCG and NCG were adopted to compute the F-measure scores (Methods) for assessing the performance of different methods. In total, we collect 616 cancer census genes and 711 known cancer genes from CCG and NCG gene lists (Additional File 2).

In the method comparisons, PNC selects the cancer driver genes which have high frequency (>0.8) among all the patients on each cancer data sets. From Figure 3, we found that the F-measures of PNC would be higher than 9 methods on gene mutation data (i.e., DriverML [7], SCS [8], DawnRank [9], ActiveDriver [22], DriverNet [23], MutSigCV [24], OncoDriveFM [25], SSN [26] and Mutation frequency method) and 4 methods on gene expression data (i.e., DEG-FoldChange, DEG-p-value, DEG-FDR and Hub genes selection method) on 13 cancer datasets. We showed the predicted driver genes list of different methods in Additional file 4. We obtained the cancer driver genes of DriverML [7], SCS[8], and SSN [26] from their provided driver genes list. We obtained the driver genes in ActiveDriver [22], OncodriverFM[25], MutSigCV[24], DriverNet[23], and DawnRank [9] from the DriverDBv2 database [34]. Besides, the DEG-FoldChange selects the personalized driver genes by calculating the fold-change between the normal sample and tumor sample (|log2(fold-change)|>1). DEG-p-value and DEG-FDR select the personalized driver genes by respectively calculating the p-value and FDR (<0.05) between a cancer tumor sample and a group of control samples. Hub genes selection method regards hub genes in the constructed network as cancer driver genes (Methods). All the above methods ran the same TCGA datasets according to their manuals.

Figure 3 The significant enrichment F-scores of PNC and other methods for identifying cancer driver genes.

*f. The authors compared the PNC with DEG-Fold and Hub gene selection, did they compare with somatic mutations, gene amplification, and copy-number changes?*

**Answer：**

We appreciate Reviewer #3 for offering us these critical methods based on somatic mutations, gene amplification, and copy-number changes. To give a comparison of PNC with current state-of-the-art methods based on mutation changes, our PNC selected the cancer driver genes which have high frequency (>0.8) among all the patients on each cancer data sets. From Figure 3, we found that the F-measures of PNC would be higher than 9 cancer driver genes focus methods on gene mutation data with somatic mutations and copy-number mutations (i.e., DriverML[17], ActiveDriver[18], DriverNet [19], MutSigCV [20], OncoDriveFM [21], SCS [22], DawnRank [14], SSN [1] and Mutation frequency-based method) on 13 cancer datasets. In the future, we will compare PNC with methods on gene amplification mutation data.

**Changes in the manuscript:**

**You may find the revised paragraph highlighted on pages 12-13 as bellow:**

To identify personalized driver genes, we collected paired samples (a normal sample and a tumor sample) from each individual. Here we used cancer datasets which contained enough normal-disease paired samples (>20 paired samples) in TCGA for the case study. By searching TCGA, 13 cancer datasets met the requirements, i.e., the datasets for breast invasive carcinoma (BRCA), colon adenocarcinoma (COAD), kidney chromophobe (KICH) and kidney renal clear cell carcinoma (KIRC), kidney renal papillary cell carcinoma (KIRP), liver hepatocellular carcinoma (LIHC), lung adenocarcinoma (LUAD), lung squamous cell carcinoma (LUSC), stomach adenocarcinoma (STAD), uterine corpus endometrial carcinoma(UCEC), Head and Neck Squamous Cell Carcinoma (HNSC), Prostate Adenocarcinoma (PRAD) and Thyroid Papillary Carcinoma (THCA). For more information, see Table S1 of Additional File 1. On the 13 cancer datasets, the identified cancer driver genes annotated in the CCG and NCG were adopted to compute the F-measure scores (Methods) for assessing the performance of different methods. In total, we collect 616 cancer census genes and 711 known cancer genes from CCG and NCG gene lists (Additional File 2).

In the method comparisons, PNC selects the cancer driver genes which have high frequency (>0.8) among all the patients on each cancer data sets. From Figure 3, we found that the F-measures of PNC would be higher than 9 methods on gene mutation data (i.e., DriverML [7], SCS [8], DawnRank [9], ActiveDriver [22], DriverNet [23], MutSigCV [24], OncoDriveFM [25], SSN [26] and Mutation frequency method) and 4 methods on gene expression data (i.e., DEG-FoldChange, DEG-p-value, DEG-FDR and Hub genes selection method) on 13 cancer datasets. We showed the predicted driver genes list of different methods in Additional file 4. We obtained the cancer driver genes of DriverML [7], SCS[8], and SSN [26] from their provided driver genes list. We obtained the driver genes in ActiveDriver [22], OncodriverFM[25], MutSigCV[24], DriverNet[23], and DawnRank [9] from the DriverDBv2 database [34]. Besides, the DEG-FoldChange selects the personalized driver genes by calculating the fold-change between the normal sample and tumor sample (|log2(fold-change)|>1). DEG-p-value and DEG-FDR select the personalized driver genes by respectively calculating the p-value and FDR (<0.05) between a cancer tumor sample and a group of control samples. Hub genes selection method regards hub genes in the constructed network as cancer driver genes (Methods). All the above methods ran the same TCGA datasets according to their manuals.

*g. In the supplementary note 2, the number of paired samples is less than 80 in 12 cancer types, with such litter number, a lot of theoretical assumptions are not true due to n is small, even n=200 is very different from the assumption . The assumption regarding the distribution of PCC, the distribution of ΔPCC and so on are not true. The sample-specific network theory can’t be applied in such a situation with limited numbers of patients.*

**Answer：**

We appreciate Reviewer #3 for this very constructive suggestion. Following Reviewer #3’s suggestion, we have added the explanation of the sample-specific network theory [1] on the situation with limited numbers of patients. To illustrate that the sample-specific network theory [1] can be applied in situations with limited numbers of patients, we generated two series of reference numbers. We choose the length *n* of the two series (i.e., the number of the reference samples) as 20, 50 and 100 and the correlation of the two series of numbers was a fixed value (*PCCn* =0). Based on the generated two series of reference numbers, we randomly generated one series of two numbers (gene expression value of two genes for one sample) and obtained the distribution of Δ*PCCn*. The random simulation was repeated 2,000,000 times, where the value of Δ*PCCn* with a significant P-value of 0.05 in the two-tails area was selected from every distribution of simulation (Figure S3 (d) of Additional file 1).

As shown in Figure S3, Reviewer #3 is correct, and the distribution of ΔPCC is not the theoretical normal distribution. Although there exist differences between the true distribution and the theoretical normal distribution, the tail area is similar to a normal distribution. Furthermore, the z-scores of Δ*PCCn* with the P-value of 0.05 for the random simulation and the theoretical calculation have very little difference (Figure S3 (d)). Therefore, SSN method [1] is suitable for the situation with limited numbers of patients. For this question, we thank Professor Liu Xiaoping from Shandong University, China (the first author of SSN method) for offering us assistance.

Figure S3 (a-c) The distribution of Δ*PCCn* numerically obtained by random simulation (*n*=20, 50 and 100). (d) The significant z-score of Δ*PCCn* evaluated from the distribution of the random simulation (blue color) and the theoretical distribution (red color).

**Changes in the manuscript:**

**You may find the revised paragraph highlighted on pages 25-26 as bellow:**

To illustrate that the sample-specific network theory can be applied in situations with limited numbers of patients, we generated two series of reference numbers. The length *n* of the two series (i.e. the number of the reference samples) was chose as 20, 50 and 100 and the correlation of the two series of numbers was a fixed value (*PCCn* =0). Based on the generated two series of reference numbers, we randomly generated one series of two numbers (gene expression value of two genes for one sample) and obtain the distribution of Δ*PCCn*. The random simulation was repeated 2,000,000 times, where the value of Δ*PCCn* with a significant P-value of 0.05 in the two-tails area was selected from every distribution of simulation (Figure S3 (d)). As shown in Figure S3 (a-c), the distribution of Δ*PCCn* follows a new type of distribution defined as volcano distribution, whose tail areas are similar to those of a normal distribution in a random condition. At the same time, the significant value of Δ*PCCn* with the P-value of 0.05 can also be obtained from Equation (2) (Figure S3 (d)). The z-scores of Δ*PCCn* with the P-value of 0.05 for the random simulation and the theoretical calculation have little difference (Figure S3 (d)). The above results well validates that SSN method can be suitable for situation with limited numbers of patients.

*h. I am not sure how the driver genes are defined?*

**Answer：**

It seems that the interpretation of the driver genes was unclear in the previous version. Following Reviewer #3’s value comments, we have provided detailed interpretation of the driver genes. Cancer is caused by somatic mutations that confer a selective advantage to cells [20,23]. During tumor progression, the majority of detected altered genes are passengers that do not contribute to oncogenic process but a small fraction of genomic and transcriptomic altered genes are known as driver genes that modify transcriptional programs and therefore drive and sustain tumor progression[17,24]. The driver genes are the small number of altered genes by the oncogene activation signals such as genetic mutation, gene amplification, chromosomal rearrangement or transposable elements which can drive the tumor progression from healthy state to disease state. Many bioinformatics tools for driver gene identification with multi-dimensional genomic data have been developed [14,17,20,22-27]. Although these methods have been successfully used for prioritizing driver genes in cancer, the ultimate goal of discovering a complete catalog of driver genes truly associated with individual patients is far from being achieved. Therefore, new and efficient computational models or methods are urgently needed to identify personalized driver genes for understanding tumor heterogeneity in cancer.

From the dynamics point of view, gene expressions are variables of such a system and may be different if measured at different time points. In contrast, it is transcriptional networks that thus result in the measured gene expression pattern of an individual patient [19].Indeed, transcriptional networks are composed of numerous nodes linked via a complex set of interactions, and the dynamical behavior are controlled by few number of driver nodes. Recent developing computational approaches based on network control principles are opening a new way to discover driver genes in cancer, particularly at an individual level. To provide new effective network control model on this timely topic, we considered the cancer progression as a network control problem, in which the expected personalized driver genes are altered genes by oncogene activation signals that can change the individual molecular network from one health state to the other disease state. From the structure based network control respective, the personalized driver genes are the altered nodes/genes in response to input signals which can trigger the state transition of the whole gene interaction network of an individual patient. The input signals may be oncogene activation signals such as a genetic mutation, gene amplification, a chromosomal rearrangement, or transposable elements. The “controllers” in PNC model for identifying personalized driver genes are the genetic or environment factors which produce the oncogene activation signals. Therefore, the biological interpretation of the PNC model is to identify personalized driver genes which can trigger the state transition of the individual system from normal attractor to disease attractor in response to oncogene activation signals.

**Changes in the manuscript:**

**You may find the revised paragraph highlighted on pages 3-4 as bellow:**

Cancer is a heterogeneous disease that is driven by oncogene activations such as genetic mutation, gene amplification, chromosomal rearrangement, and transposable elements [1-3]. During tumor progression, the majority of detected altered genes are passengers that do not contribute to the oncogenic process, but a small fraction of genomic and transcriptomic altered genes are known as driver genes that modify transcriptional programs and therefore drive and sustain tumor progression from a healthy state to disease state. Many bioinformatics tools for driver gene identification with multi-dimensional genomic data have been developed recently [4-9]. To our best understanding, we categorized these approaches into two groups according to their significant features. (i) The driver gene identification methods in large cohorts, such as mutation frequency-based methods and machine learning-based methods. The mutation frequency-based methods mainly identify the drive genes by finding significantly mutated genes whose mutation rates are significantly higher than the background mutation rate [4,5]. Due to the heterogeneity of tumors, constructing a reliable background mutation model is difficult, which limits the performance of frequency-based methods. On the other hand, the machine learning-based methods can be developed for any specific tasks depending on the available training data designed as pathogenic or neutral, but they have a few applications due to the probable incompleteness of their referred databases [6, 7]. (ii) The driver gene identification methods for individual patients. These methods such as SCS [8], DawnRank [9] assume that cancer is a complex disease with many changes altered at the network level and identify personalized driver genes by integrating personalized genetic data of an individual patient and the reference gene (or protein) interaction data. Although these methods have been successfully used for prioritizing cancer driver genes, the ultimate goal of discovering a complete catalog of driver genes indeed associated with individual patients is far from being achieved. Therefore, new and efficient computational models and methods are urgently needed to identify personalized driver genes for understanding tumor heterogeneity in cancer.

**You may find the revised paragraph highlighted on pages 10-11 as bellow:**

The biological system in an individul patient is generally a nonlinear dynamical system. From the dynamics point of view, gene expressions are variables of such a system and may be different if measured at different time points. In contrast, it is transcriptional networks that thus result in the measured gene expression pattern and determine the state transition of an individual patient in caner development [23]. The state transition networks or transcriptional networks of an individul patient can more reliably characterize the biological system of the individual patient. The biological interpretation of personalized state transition network represents which gene pairs are involved in the disease development for each patient.

It is important to reconstruct the personalized state transition networks with the personalized genetic data (e.g., expression profiles). Currently most of the studies for exploiting gene regulation, such as the Single Sample Network (SSN) [26] and Linear Interpolation to Obtain Network Estimates for Single Samples(LIONESS) [27], can describe the dynamic gene regulation for single tumor sample of an individual patient. But they ignore normal sample information of an individual patient and consequently and they yield many false-positive calls for constructing personalized state transition network. Therefore to construct the personalized state transition network for each patient, we designed Paired-SSN method based on sample specific network theory (SSN) [26] for characterizing significant difference of gene pairs between normal sample and tumor sample of an individual patient. In Figure 2, we gave some examples how to apply Paired-SSN for constructing the personalized state transition network based on the expression data of two cancer patients (TCGA.G9.6499 and TCGA.EJ.7781). Then based on the structure of personalized state transition network, we developed NCUA method based structure based network control theory [10-13] to identify driver genes for driving the whole network state of an individual patient from the normal attractor to disease attractor. From the structure based network control respective, the personalized driver genes are the altered nodes/genes in response to input signals which can trigger the state transition of the whole network of an individual patient. The input signals may be oncogene activation signals such as a genetic mutation, gene amplification, a chromosomal rearrangement, or transposable elements. The “controllers” in PNC model for identifying personalized driver genes are the genetic or environment factors which produce the oncogene activation signals. Therefore the biological interpretation of the PNC model is to identify personalized driver genes which can trigger the state transition of the individual system from normal attractor to disease attractor in response to oncogene activation signals.

*i. There is no time-serious data, I am not sure how many states does the nodes have in the personalized state transition networks, how to determine the transition states? How to characterize the state transition of each individual in the PNC? Also in the supplementary file, in equation s2, t→∞, I am not sure what the t represents physically? As all the data are static.*

**Answer：**

Thanks for the reviewer’s valuable comments. It seems that some sentences for explaining how to characterize and determine the personalized state transition networks in PNC model are missing. Therefore, we added more sentences to explain how to characterize and determine the personalized state transition networks in PNC model.

The biological system in an individul patient is generally a nonlinear dynamical system. In contrast, it is transcriptional networks that thus result in the measured gene expression pattern and determine the state transition of an individual patient in caner development [19]. The state transition networks or transcriptional networks of an individul patient can more reliably characterize the biological system of the individual patient. In the personalized state transition networks, there exist states in the normal attractor and disease attractor. The PNC aims to identify the driver nodes which can drive the state transition from the normal attractor to the disease attractor.

Reviewer #3 is correct, and it is important to shown how to determine the personalized state transition networks with the personalized genetic data (e.g., expression profiles). Currently most of the studies for exploiting gene regulation, such as the Single Sample Network (SSN) [3] and Linear Interpolation to Obtain Network Estimates for Single Samples(LIONESS) [12], can describe the dynamic gene regulation for single tumor sample of an individual patient. But they ignore normal sample information of an individual patient and consequently, they yield many false-positive calls for constructing personalized state transition network. To construct the personalized state transition network in the disease development for each patient, we designed Paired-SSN method for characterizing significant difference of gene pairs between normal sample and tumor sample of an individual patient. For a given cancer dataset, we chose the expression data of all normal samples as the reference data and respectively constructed the co-expression network of tumor sample and normal sample with the reference data by using SSN method. Then the personalized state transition network was constructed where the nodes represent the genes and edges will exist if p-value of the gene interaction edge is less than (greater than) 0.05 in the tumor sample network but greater than (less than) 0.05 in the normal sample network.

We do agree that the physical interpretation of *t* in Equation S2 was not explained clearly in the previous version of our manuscript. In Equation S2 of Additional file 1, *x_i_*(*t*) denotes the state of the variable associated to node *i* at time *t* during the period from initial attractor to disease attractor. Equation S2 denotes that for all choices of nonlinearities function if and only if we drive the state of a feedback vertex set (FVS) of the undirected graph from the initial state to desired attractor (*t*→∞, *x_J_*(*t*)→$\mathop{x}\limits^{\text{\textasciitilde}}$*_J_*(*t*)), the whole network state will be changed from an initial attractor to a desired attractor (*t*→∞, *x* (*t*)→$\mathop{x}\limits^{\text{\textasciitilde}}$(*t*)). The *x* and $\mathop{x}\limits^{\text{\textasciitilde}}$ denotes the state variable in the normal attractor and disease attractor of personalized state transition networks respectively.

**Changes in the manuscript:**

**You may find the revised paragraph highlighted on pages 10-11 as bellow:**

The biological system in an individul patient is generally a nonlinear dynamical system. From the dynamics point of view, gene expressions are variables of such a system and may be different if measured at different time points for the same patient. In contrast, it is transcriptional networks that thus result in the measured gene expression pattern and determine the state transition of an individual patient in caner development [23]. The state transition networks or transcriptional networks of an individul patient can more reliably characterize the biological system of the individual patient. The biological interpretation of personalized state transition network represents which gene pairs are involved in the disease development for each patient.

Thus it is important to reconstruct the personalized state transition networks with the personalized genetic data (e.g., expression profiles). Currently most of the studies for exploiting gene regulation, such as the Single Sample Network (SSN) [26] and Linear Interpolation to Obtain Network Estimates for Single Samples(LIONESS) [27], can describe the dynamic gene regulation for single tumor sample of an individual patient. But they ignore normal sample information of an individual patient and consequently and they yield many false-positive calls for constructing personalized state transition network. Therefore to construct the personalized state transition network for each patient, we designed Paired-SSN method based on sample specific network theory (SSN) [26] for characterizing significant difference of gene pairs between normal sample and tumor sample of an individual patient. In Figure 2, we gave some examples how to apply Paired-SSN for constructing the personalized state transition network based on the expression data of two cancer patients (TCGA.G9.6499 and TCGA.EJ.7781).

Then based on the structure of personalized state transition network, we developed NCUA method based structure based network control theory [10-13] to identify driver genes for driving the whole network state of an individual patient from the normal attractor to disease attractor. From the structure based network control respective, the personalized driver genes are the altered nodes/genes in response to input signals which can trigger the state transition of the whole gene interaction network of an individual patient. The input signals may be oncogene activation signals such as a genetic mutation, gene amplification, a chromosomal rearrangement, or transposable elements. The “controllers” in PNC model for identifying personalized driver genes are the genetic or environment factors which produce the oncogene activation signals. Therefore the biological interpretation of the PNC model is to identify personalized driver genes which can trigger the state transition of the individual system from normal attractor to disease attractor in response to oncogene activation signals.

**You may find the revised paragraph highlighted on page 2 of Additional file 1 as bellow:**

*Then any two solutions x and* $\mathop{x}\limits^{\text{\textasciitilde}}$ *of Eq. S1* *satisfy*

$\begin{matrix} \lim_{t\to\infty} \left(x_{J}(t)-\mathop{x}\limits^{\text{\textasciitilde}} _{J}(t)\right)\to0 \\ \Rightarrow\lim_{t\to\infty} \left(x(t)-\mathop{x}\limits^{\text{\textasciitilde}} (t)\right)\to0 \end{matrix}$ (S2)

*for all choices of nonlinearities*$F_{i}$*if and only if J is a feedback vertex set (FVS) of the undirected graph G.*

In Equation (S2), *x_i_*(*t*) denotes the state of the variable associated to node *i* at time *t* from initial attractor to disease attractor. The two solutions *x* and $\mathop{x}\limits^{\text{\textasciitilde}}$ denotes the state variable in the normal attractor and disease attractor of personalized state transition networks respectively. Equation S2 denotes that for all choices of nonlinearities function if and only if we drive the state of a feedback vertex set (FVS) of the undirected graph from the initial state to desired attractor (*t*→∞, *x_J_*(*t*)→$\mathop{x}\limits^{\text{\textasciitilde}} _{J}(t)$), the whole network state will be changed from an initial attractor to a desired attractor (*t*→∞, *x* (*t*)→$\mathop{x}\limits^{\text{\textasciitilde}} (t)$).

*j. In Table S2, the NCUA is an NP-hard problem, i.e., every time one performs the NUCA, and the result can be different.*

**Answer：**

It seems that the previous description in the original manuscript was unclear, which leads to a misunderstanding of our method. Please allow us to explain it clearly here. The NP-hard ([non-deterministic polynomial-time](https://en.wikipedia.org/wiki/NP_(complexity)) hard) problem, in [computational complexity theory](https://en.wikipedia.org/wiki/Computational_complexity_theory) is defined as: *a problem H is NP-hard when every problem L in NP can be* [*reduced*](https://en.wikipedia.org/wiki/Reduction_(complexity)) *in* [*polynomial time*](https://en.wikipedia.org/wiki/Polynomial_time) *to H; that is, assuming a solution for H takes 1 unit time, we can use H‎'s solution to solve L in polynomial time* [[28]](https://en.wikipedia.org/wiki/NP-hardness#cite_note-Leeuwen-1). As a consequence, finding a polynomial algorithm to solve any NP-hard problem would give polynomial algorithms for all the problems in NP, which is unlikely as many of them are considered difficult [[28]](https://en.wikipedia.org/wiki/NP-hardness#cite_note-3). Although it is suspected that there are no polynomial-time algorithms for NP-hard problems, this has not been proven.

Although NUCA is an NP-hard problem, the optional solution can be obtained for the moderate size graphs with up to a few tens of thousands of nodes by utilizing the LP-based classic branch and bound method [29].

To summarize, every time one performs the PNC, the result is NOT different. We have uploaded the PNC code to website <https://github.com/NWPU-903PR/PNC>.

**Changes in the manuscript:**

**You may find the revised paragraph highlighted on page 30 as bellow:**

The NP-hard problem, in [computational complexity theory](https://en.wikipedia.org/wiki/Computational_complexity_theory) is defined as: *a problem H is NP-hard when every problem L in NP can be* [*reduced*](https://en.wikipedia.org/wiki/Reduction_(complexity)) *in* [*polynomial time*](https://en.wikipedia.org/wiki/Polynomial_time) *to H; that is, assuming a solution for H takes 1 unit time, we can use H‎'s solution to solve L in polynomial time* [[49]](https://en.wikipedia.org/wiki/NP-hardness#cite_note-Leeuwen-1). As a consequence, finding a polynomial algorithm to solve any NP-hard problem would give polynomial algorithms for all the problems in NP, which is unlikely as many of them are considered difficult [[49]](https://en.wikipedia.org/wiki/NP-hardness#cite_note-Leeuwen-1). Although it is an NP-hard problem, the optional solution can be obtained for the moderate size graphs with up to a few tens of thousands of nodes by utilizing the LP-based classic branch and bound method [48].

**You may find the revised paragraph highlighted on page 33 as bellow:**

**Availability of code and datasets**

The PNC code and the data resources used in this work can be freely downloaded from <https://github.com/NWPU-903PR/PNC>.

**Reference**

1. Liu X, Wang Y, Ji H, Aihara K, Chen L (2016) Personalized characterization of diseases using sample-specific networks. Nucleic Acids Research 44: e164-e164.

2. Kuijjer ML, Tung MG, Yuan G, Quackenbush J, Glass K (2019) Estimating sample-specific regulatory networks. iScience 14: 226-240.

3. Cornelius SP, Kath WL, Motter AE (2013) Realistic control of network dynamics. Nature communications 4: 1942.

4. Wang L-Z, Su R-Q, Huang Z-G, Wang X, Wang W-X, et al. (2016) A geometrical approach to control and controllability of nonlinear dynamical networks. Nature communications 7: 11323.

5. Karl S, Dandekar T (2015) Convergence behaviour and control in non-linear biological networks. Scientific Reports 5: 9746.

6. Mochizuki A, Fiedler B, Kurosawa G, Saito D (2013) Dynamics and control at feedback vertex sets. II: A faithful monitor to determine the diversity of molecular activities in regulatory networks. Journal of theoretical biology 335: 130-146.

7. Fiedler B, Mochizuki A, Kurosawa G, Saito D (2013) Dynamics and control at feedback vertex sets. I: Informative and determining nodes in regulatory networks. Journal of Dynamics and Differential Equations 25: 563-604.

8. Zañudo JGT, Yang G, Albert R (2017) Structure-based control of complex networks with nonlinear dynamics. Proceedings of the National Academy of Sciences 114: 7234-7239.

9. Liu Y, Slotine JJ, Barabasi AL. Controllability of Complex Networks; 2011.Nature.

10. Ciriello G, Cerami E, Sander C, Schultz N (2012) Mutual exclusivity analysis identifies oncogenic network modules. Genome research 22: 398-406.

11. Croft D, O’Kelly G, Wu G, Haw R, Gillespie M, et al. (2010) Reactome: a database of reactions, pathways and biological processes. Nucleic Acids Research 39: D691-D697.

12. Schaefer CF, Anthony K, Krupa S, Buchoff J, Day M, et al. (2008) PID: the pathway interaction database. Nucleic Acids Research 37: D674-D679.

13. Kanehisa M, Goto S, Sato Y, Furumichi M, Tanabe M (2011) KEGG for integration and interpretation of large-scale molecular data sets. Nucleic Acids Research 40: D109-D114.

14. Hou JP, Ma J (2014) DawnRank: discovering personalized driver genes in cancer. Genome medicine 6: 56.

15. Hu Y, Chen C-h, Ding Y-y, Wen X, Wang B, et al. (2019) Optimal control nodes in disease-perturbed networks as targets for combination therapy. Nature communications 10: 2180.

16. Quan Y, Liu M-Y, Liu Y-M, Zhu L-D, Wu Y-S, et al. (2018) Facilitating anti-cancer combinatorial drug discovery by targeting epistatic disease genes. Molecules 23: 736.

17. Han Y, Yang J, Qian X, Cheng W-C, Liu S-H, et al. (2019) DriverML: a machine learning algorithm for identifying driver genes in cancer sequencing studies. Nucleic Acids Research 47: e45-e45.

18. Reimand J, Bader GD (2013) Systematic analysis of somatic mutations in phosphorylation signaling predicts novel cancer drivers. Molecular systems biology 9.

19. Bashashati A, Haffari G, Ding J, Ha G, Lui K, et al. (2012) DriverNet: uncovering the impact of somatic driver mutations on transcriptional networks in cancer. Genome biology 13: R124.

20. Lawrence MS, Stojanov P, Polak P, Kryukov GV, Cibulskis K, et al. (2013) Mutational heterogeneity in cancer and the search for new cancer-associated genes. Nature 499: 214.

21. Gonzalez-Perez A, Lopez-Bigas N (2012) Functional impact bias reveals cancer drivers. Nucleic Acids Research 40: e169-e169.

22. Guo W-F, Zhang S-W, Liu L-L, Liu F, Shi Q-Q, et al. (2018) Discovering personalized driver mutation profiles of single samples in cancer by network control strategy. Bioinformatics 34: 1893-1903.

23. Tamborero D, Gonzalez-Perez A, Lopez-Bigas N (2013) OncodriveCLUST: exploiting the positional clustering of somatic mutations to identify cancer genes. Bioinformatics 29: 2238-2244.

24. Luo P, Ding Y, Lei X, Wu F-X (2019) deepDriver: predicting cancer driver genes based on somatic mutations using deep convolutional neural networks. Frontiers in genetics 10.

25. Bertrand D, Chng KR, Sherbaf FG, Kiesel A, Chia BK, et al. (2015) Patient-specific driver gene prediction and risk assessment through integrated network analysis of cancer omics profiles. Nucleic Acids Research 43: e44-e44.

26. Boccaletti S, Latora V, Moreno Y, Chavez M, Hwang D-U (2006) Complex networks: Structure and dynamics. Physics reports 424: 175-308.

27. Zeng T, Zhang W, Yu X, Liu X, Li M, et al. (2015) Big-data-based edge biomarkers: study on dynamical drug sensitivity and resistance in individuals. Briefings in bioinformatics 17: 576-592.

28. Rayward-Smith, V. J. (1995). Introduction to the Theory of Complexity. journal of the Operational Research Society, 46(12), 1507-1508.

29. Lenstra Jr HW (1983) Integer programming with a fixed number of variables. Mathematics of operations research 8: 538-548.

30.Takahashi K, et al. (2007) Induction of pluripotent stem cells from adult human fibroblasts by defined factors. Cell 131:861–872.
